# Proton Pump Inhibitor Use and Survival in Patients With Newly Diagnosed Glioblastoma

**DOI:** 10.1001/jamanetworkopen.2025.45578

**Published:** 2025-11-25

**Authors:** Emilie Le Rhun, Debaleena Sain, Sara C. Erridge, David A. Reardon, Giuseppe Minniti, Patrick Roth, Wolfgang Wick, Burt Nabors, John Sampson, Warren Mason, Tim Cloughesy, Jacob C. Reijneveld, Roger Stupp, Matthias Preusser, Thierry Gorlia, Michael Weller

**Affiliations:** 1 Department of Medical Oncology and Hematology, University Hospital and University of Zurich, Zurich, Switzerland; 2 Department of Neurology, University Hospital and University of Zurich, Zurich, Switzerland; 3 European Organisation for Research and Treatment of Cancer Headquarters, Brussels, Belgium; 4 Edinburgh Cancer Centre, University of Edinburgh, Edinburgh, United Kingdom; 5 Center for Neuro-Oncology, Dana-Farber Cancer Institute, Harvard Medical School, Boston, Massachusetts; 6 Department of Radiological Sciences, Oncology and Anatomical Pathology, Sapienza University of Rome, Rome, Italy; 7 Policlinico Umberto I, Rome, Italy; 8 Department of Neurology and European Center for Neurooncology, University Hospital Heidelberg, Heidelberg University, Heidelberg, Germany; 9 Department of Neurology, University of Alabama at Birmingham, Birmingham; 10 Department of Neurosurgery, Duke University Medical Center, Preston Robert Tisch Brain Tumor Center at Duke, Durham, North Carolina; 11 Department of Medicine, Princess Margaret Cancer Centre, University of Toronto, Toronto, Ontario, Canada; 12 Department of Neurology, David Geffen School of Medicine, University of California, Los Angeles; 13 Department of Neurology & Brain Tumor Center Amsterdam, Amsterdam University Medical Center, Amsterdam, the Netherlands; 14 Department of Neurology, Stichting Epilepsie Instellingen Nederland, Heemstede, the Netherlands; 15 Lou and Jean Malnati Brain Tumor Institute, Robert H. Lurie Comprehensive Cancer Center, Feinberg School of Medicine, Northwestern University, Chicago, Illinois; 16 Division of Oncology, Department of Medicine I, Medical University of Vienna, Vienna, Austria; 17 Clinical Cooperation Unit Neuro-Oncology, German Cancer Research Center and German Consortium for Translational Cancer Research, Heidelberg, Germany

## Abstract

**Importance:**

Proton pump inhibitors (PPI) are often prescribed to prevent steroid-induced gastritis and peptic ulcer disease in patients with glioblastoma. Yet, these drugs may enhance the activity of aldehyde dehydrogenase 1 A1 (ALDH1A1), which has been linked to protection from oxidative stress, radiotherapy, and chemotherapy.

**Objective:**

To explore the associations of the use of potent ALDH1A1-activating PPIs (PA-PPIs) and other antacid drugs with outcomes in patients with newly diagnosed glioblastoma.

**Design, Setting, and Participants:**

This meta-analysis was a secondary analysis of individual prospectively captured patient data using a dataset of 5 randomized clinical trials conducted between 2008 and 2020. Participants included patients with a new diagnosis of glioblastoma. Data analysis was completed in November 2024.

**Exposure:**

We assessed drug use at baseline and defined 3 landmarks: start of temozolomide maintenance cycles 1 (landmark 1) and 4 (landmark 2), and end of cycle 6 (landmark 3).

**Main Outcomes and Measures:**

The primary outcome measures were progression-free survival (PFS) and overall survival (OS) from baseline and from the start of each corresponding landmark time.

**Results:**

The study population included 2981 patients (1858 [62.3%] male; median [range] age, 58 [18-85] years). On univariate analysis, patients treated with PA-PPI had worse PFS and OS at all 4 time points. The multivariate analysis accounting for age, sex, performance status, steroid use, extent of resection, and *MGMT* status confirmed a difference for PFS at landmarks 1 (hazard ratio [HR], 1.14 [95% CI, 1.01-1.28]), 2 (HR, 1.26 [95% CI, 1.09-1.44]), and 3 (HR, 1.31 [95% CI, 1.10-1.56]), and for OS at landmarks 1 (HR, 1.34 [95% CI, 1.08-1.66]) and 2 (HR, 1.14 [95% CI, 1.01-1.29]). No such association was seen for the use of other antacid drugs. The negative association of PA-PPI use with PFS and OS was observed independently of *MGMT* promoter methylation and steroid use.

**Conclusions and Relevance:**

In this meta-analysis of patients with newly diagnosed glioblastoma, PPI use was associated with inferior survival outcomes. These findings suggest that PA-PPI use should be discouraged in patients with glioblastoma, since alternative agents are available and a detrimental effect cannot be excluded. Translational research studies should explore whether PPI-induced activity of ALDH mediates the potential adverse effects of PPI in glioblastoma.

.

## Key Points

**Question:**

Is exposure of patients with newly diagnosed glioblastoma to proton pump inhibitors associated with survival outcomes?

**Findings:**

This meta-analysis of individual data from 2981 patients enrolled into 5 prospective clinical trials found that the use of proton pump inhibitors that are potent activators of aldehyde dehydrogenase 1 A1 was associated with inferior survival outcomes, independently of *MGMT* promoter methylation status and steroid use. No such association was observed with other antacid medications.

**Meaning:**

These findings suggest that PA-PPI use should be discouraged in patients with glioblastoma.

## Teaser

This meta-analysis explores the associations of the use of potent ALDH1A1-activating proton pump inhibitors and other antacid drugs with survival outcomes in patients with newly diagnosed glioblastoma.

## Introduction

Proton pump inhibitors (PPIs) are often used in neurooncology to reduce the risk of gastritis and peptic ulcer disease when steroids are prescribed to control mass effect and cerebral edema. There has been increasing interest in disease- or treatment-modifying effects of PPIs in patients with cancer because PPIs alter the pH of the micromilieu and may affect cancer biology and treatment efficacy.^1^ Furthermore, preclinical studies demonstrate that pH alterations may affect sensitivity of glioblastoma cells to irradiation or chemotherapy.^2^ Studies on the effects of PPI exposure in various in vitro or in vivo cancer settings have yielded different outcomes. Pretreatment with PPI of cell lines derived from melanomas, adenocarcinomas, and lymphomas sensitized to cisplatin, 5-fluorouracil, and vinblastine in vitro and oral pretreatment with omeprazole induced sensitivity of human xenograft solid tumors to cisplatin in vivo.^3^ Furthermore, an inhibition of invasion in vitro and of tumor growth in vivo after omeprazole treatment has been reported in a glioblastoma model.^4^ Rabeprazole has been reported to inhibit glioma cell growth and sensitize to temozolomide by repressing the epithelial-to-mesenchymal transition, mediated by impeding Ak strain transforming or glycogen synthase kinase 3β phosphorylation and nuclear factor κB signaling.^5^ Conversely, PPI use has been associated with inferior outcomes in patients with non–small cell lung cancer,^6^ patients with cancer treated with immune checkpoint inhibitors in general,^7^ and patients with glioblastoma based on an analysis of electronic medical records.^8^ The commonly used drugs omeprazole and pantoprazole have been reported as potent activators of the enzymatic activity of human aldehyde dehydrogenases 1 A1 (ALDH1A1), whereas lansoprazole and rabeprazole (substitution in the pyridine ring) were reported as less active.^9^ Members of the ALDH superfamily that includes 19 genes in humans are responsible for detoxification of lipid aldehydes generated during oxidative stress, and ALDH1A1 has been specifically linked to cancer stemness.^10^ Cellular protection from oxidative stress by omeprazole was blocked by inhibition of ALDH with disulfiram.^11^ Based on putative *MGMT*-depleting properties of the bona fide ALDH1 inhibitor, disulfiram, and its sensitization to alkylating chemotherapy,^12^ clinical studies have already started to explore the role of disulfiram in patients with glioblastoma,^13^ although a first trial of adding disulfiram and copper to standard care chemotherapy in patients with recurrent glioblastoma showed no evidence of activity.^14^

Given these data, we became intrigued by the possibility that the detrimental association of steroid use with survival outcomes in glioblastoma^15^ might be mediated in part or entirely by the prescription of PPIs to counteract adverse effects of steroids. We performed a pooled analysis of randomized clinical trials in patients with newly diagnosed glioblastoma to test the hypothesis that PPI use is associated with survival outcomes in patients with glioblastoma and to derive a theoretical framework of cause and effects of such an association.

## Methods

This meta-analysis was approved by the Cantonal Ethics Committee in Zurich, Switzerland, with a waiver of informed consent because of the use of secondary data. The analysis followed the Preferred Reporting Items for Systematic Reviews and Meta-Analyses (PRISMA) reporting guideline.

### Study Design

The main objective of this study was to explore the associations of PPI use with progression-free survival (PFS) and overall survival (OS) in patients with newly diagnosed glioblastoma. Secondary objectives were to investigate survival outcomes associated with PPI use by *MGMT*-promoter methylation status and by steroid use. To assess the association between PPI intake and outcome, pooled analyses were performed for 5 clinical trials in patients with newly diagnosed glioblastoma: ACT IV,^16^ AVAglio,^17^ CENTRIC,^18^ CORE,^19^ and EORTC 1709^20^ (eTable 1 and eFigure 1 in Supplement 1).

Patients with known isocitrate dehydrogenase mutation or 1p/19q codeletion were excluded from the analysis. In all trials, data on PPI administration were collected using the concomitant medication forms.

Medications altering gastric pH were classified into (1) PPI that are potent activators of ALDH1A1 (PA-PPI) (eg, omeprazole, pantoprazole), (2) PPI that are weak activators of ALDH1A1 (eg, lansoprazole, rabeprazole), (3) systemic H2-blocker antacids (eg, cimetidine, ranitidine), and (4) locally acting antacids (eg, aluminum/magnesium antacids) (eTable 2 in Supplement 1). Given that PPI use depends on the period of assessment, a landmark analysis approach was used.^21,22^ We evaluated PPI use at start of study (baseline) and at key time points thereafter: 70 days later (landmark 1: start of maintenance cycle 1), 84 days after that (landmark 2: start of cycle 4), and another 84 days later (landmark 3: end of cycle 6). If patients discontinued treatment between 2 landmarks but had follow-up information on PPI intake, steroid use, and World Health Organization (WHO) performance status, we calculated theoretical landmark dates based on their last available data. These patients remained in the analysis as long as they did not experience progression or death or were not censored before the next theoretical landmark date for PFS and did not die or were not censored for OS analysis.

Baseline was defined as the date of enrollment, except for ACT IV,^16^ as this trial randomized patients after concomitant chemoradiotherapy. Therefore, PFS and OS in ACT IV were calculated from the first date of receiving study drug and ACT IV was excluded from analyses at baseline.

PFS and OS were measured from baseline and from each landmark. PFS was defined as the time from baseline to either disease progression or death from any cause, whichever came first. If a patient was alive and disease free at the time of analysis, PFS was censored at their last disease assessment. Progression was mainly based on imaging, although details varied over time, following the evolution of the Response Assessment in Neuro-Oncology criteria. OS was defined as the time from baseline to death from any cause, and patients who were still alive at the time of analysis were censored at the last date they were known to be alive.

### Statistical Analysis

Patients’ characteristics at baseline, overall and by trial, were described by frequencies and percentages. All outcome analyses were conducted after stratifying patients by trials, except for AVAglio,^17^ which was split into 2 strata for the 2 treatment groups, control vs bevacizumab, because of the difference in PFS. To explore the association of drug use with PFS or OS, univariate analyses were first performed through Kaplan-Meier survival plots, log-rank tests, and unadjusted Cox proportional hazards models. Next, to draw valid conclusions about these associations, multivariate Cox proportional hazards models were fitted, adjusting for potential prognostic confounding factors: age (as a continuous variable), sex (male or female), WHO performance status (PS = 0 or PS>0), steroid use (yes or no), *MGMT*-promoter status (unmethylated, methylated, or unknown), and extent of resection (complete resection or partial resection/biopsy only). At baseline and each landmark, estimates of the median survival times were reported.

For each Cox regression model, the proportional hazards assumption was assessed for drug use (the variable of interest) at each landmark using the supremum test. If the proportional hazards assumption was violated at the given level of significance (same as the assigned level for primary analysis at each time point), an interaction of drug use with time was included in the analysis. The proportional hazards assumptions were not assessed for the other adjusted factors, as they are known prognostic factors and not of specific interest.

For some patients, the start and end dates of drug administration were missing. If a patient did not have a start date for drug intake but had an end date, we assumed that they had started taking the drug prior to the baseline. Similarly, if patients had a start date but no end date and were flagged as ongoing, these patients were assumed to have continued taking these medications until the last collection date of concomitant medications (according to the protocol of the corresponding trial). For patients with both dates missing but an ongoing administration available, we assumed that the drug administration started prior to the baseline and was continued until the last date of data collection. However, if both dates were missing and no ongoing indication was available, the assumption was that those patients were not given any drugs of interest throughout the study period. It is worth noting that if data collection stopped due to progression, we had informative missingness. The proportion of patients for whom drug information was not collected at each landmark was captured. If the percentage of missing information was less than 5%, the analysis was continued ignoring the missing data (ie, complete-case analysis). If the proportion was larger than 5% but less than 15%, we performed both complete-case analysis and multiple imputation analysis (eAppendix 1 in Supplement 1). The number of imputations was equal to the next integer closest to the percentage of missingness.

To examine whether the associations of drug use with survival outcomes differ by *MGMT* promoter methylation status, forest plots and Kaplan-Meier survival plots were obtained separately for patients with *MGMT* promoter-methylated vs *MGMT* promoter-unmethylated tumors at baseline and at each landmark. Interaction tests were conducted between drug use and *MGMT* status. To investigate the association of PPI intake with the outcome associations of steroid use, forest plots were similarly obtained and interaction tests were conducted at each time point.

*P* values were 2-sided, and statistical significance for the primary analysis was established for inference at a level of 5%, which was split into 1.25% for the baseline drug analysis and 1.25% for the analysis at each of the 3 landmark times. For the analysis of all other secondary objectives, 1.25% was assigned at each time point as a screening threshold to explore interesting results. Data were analyzed using SAS version 9.4 (SAS Institute), and analysis was completed in November 2024.

## Results

### Association of PPI Use With PFS and OS

The study population included 2981 patients (1858 [62.3%] male; median [range] age, 58 [18-85] years). Patient characteristics at baseline and the major outcomes per trial are summarized in eTable 3 in Supplement 1.

The overall frequency of use of the drugs of interest in the 5 clinical trials is summarized in eTable 4 in Supplement 1, and drug use per landmark and trial is shown in Table 1. Overall, there were no significant differences in drug use across these trials. Throughout the disease course, patients with gastric symptoms documented in the case report forms had higher exposure to PPIs or other antacid drugs than patients who did not receive these drugs (eTable 5 in Supplement 1).

**Table 1.** Frequency of Use of Drug of Interest at Baseline and at Each Landmark

Drug categories	Patients by trial, No. (%)						
	ACT IV^16^	AVAglio^17^	CENTRIC^18^	CORE^19^	EORTC 1709^20^	All
**Baseline, PFS and OS**							
Overall	NA	880 (100)	371 (100)	189 (100)	707 (100)	2147 (100)
None	NA	376 (42.7)	222 (59.8)	130 (68.8)	420 (59.4)	1148 (53.5)
All PA-PPI combinations	NA	344 (39.1)	111 (29.9)	36 (19.0)	226 (32.0)	717 (33.4)
All other antacid combinations	NA	160 (18.2)	38 (10.2)	23 (12.2)	61 (8.6)	282 (13.1)
**Landmark 1, PFS** ^a^							
Overall	717 (100)	771 (100)	314 (100)	152 (100)	529 (100)	2483 (100)
None	397 (55.4)	289 (37.5)	119 (37.9)	57 (37.5)	252 (47.6)	1114 (44.9)
All PA-PPI combinations	222 (31.0)	363 (47.1)	158 (50.3)	60 (39.5)	232 (43.9)	1035 (41.7)
All other antacid combinations	98 (13.7)	119 (15.4)	37 (11.8)	35 (23.0)	45 (8.5)	334 (13.5)
**Landmark 2, PFS** ^b^							
Overall	548 (100)	623 (100)	230 (100)	94 (100)	373 (100)	1868 (100)
None	288 (52.6)	306 (49.1)	106 (46.1)	40 (42.6)	193 (51.7)	933 (49.9)
All PA-PPI combinations	188 (34.3)	243 (39.0)	99 (43.0)	38 (40.4)	153 (41.0)	721 (38.6)
All other antacid combinations	72 (13.1)	74 (11.9)	25 (10.9)	16 (17.0)	20 (5.4)	207 (11.1)
Not collected	0	0	0	0	7 (1.9)	7 (0.4)
**Landmark 3, PFS** ^c^							
Overall	400 (100)	452 (100)	185 (100)	40 (100)	242 (100)	1319 (100)
None	211 (52.8)	261 (57.7)	104 (56.2)	19 (47.5)	127 (52.5)	722 (54.7)
All PA-PPI combinations	130 (32.5)	143 (31.6)	65 (35.1)	13 (32.5)	89 (36.8)	440 (33.4)
All other antacid combinations	59 (14.8)	48 (10.6)	16 (8.6)	8 (20.0)	16 (6.6)	147 (11.1)
Not collected	0	0	0	0	10 (4.1)	10 (0.8)
**Landmark 1, OS** ^a^							
Overall	727 (100)	863 (100)	364 (100)	181 (100)	695 (100)	2830 (100)
None	402 (55.3)	313 (36.3)	135 (37.1)	67 (37.0)	318 (45.8)	1235 (43.6)
All PA-PPI combinations	226 (31.1)	414 (48.0)	184 (50.5)	74 (40.9)	317 (45.6)	1215 (42.9)
All other antacid combinations	99 (13.6)	136 (15.8)	45 (12.4)	40 (22.1)	60 (8.6)	380 (13.4)
**Landmark 2, OS** ^b^							
Overall	707 (100)	789 (100)	330 (100)	164 (100)	638 (100)	2628 (100)
None	368 (52.1)	370 (46.9)	141 (42.7)	67 (40.9)	310 (48.6)	1256 (47.8)
All PA-PPI combinations	246 (34.8)	319 (40.4)	152 (46.1)	65 (39.6)	262 (41.1)	1044 (39.7)
All other antacid combinations	93 (13.2)	99 (12.5)	35 (10.6)	30 (18.3)	47 (7.4)	304 (11.6)
Not collected	0	1 (0.1)	2 (0.6)	2 (1.2)	19 (3.0)	24 (0.9)
**Landmark 3, OS** ^c^							
Overall	675 (100)	749 (100)	303 (100)	145 (100)	597 (100)	2469 (100)
None	366 (54.2)	386 (51.5)	156 (51.5)	61 (42.1)	298 (49.9)	1267 (51.3)
All PA-PPI combinations	221 (32.7)	252 (33.6)	103 (34.0)	47 (32.4)	178 (29.8)	801 (32.4)
All other antacid combinations	88 (13.0)	81 (10.8)	19 (6.3)	19 (13.1)	27 (4.5)	234 (9.5)
Not collected	0	30 (4.0)	25 (8.3)	18 (12.4)	94 (15.7)	167 (6.8)

Abbreviations: PA-PPI, potent aldehyde dehydrogenase 1 A1 activating proton pump inhibitor; NA, not applicable; OS, overall survival; PFS, progression-free survival.

^a^ Landmark 1 was defined as the start of maintenance cycle 1.

^b^ Landmark 2 was defined as the start of maintenance cycle 4.

^c^ Landmark 3 was defined as the end of maintenance cycle.

**Figure 1.**
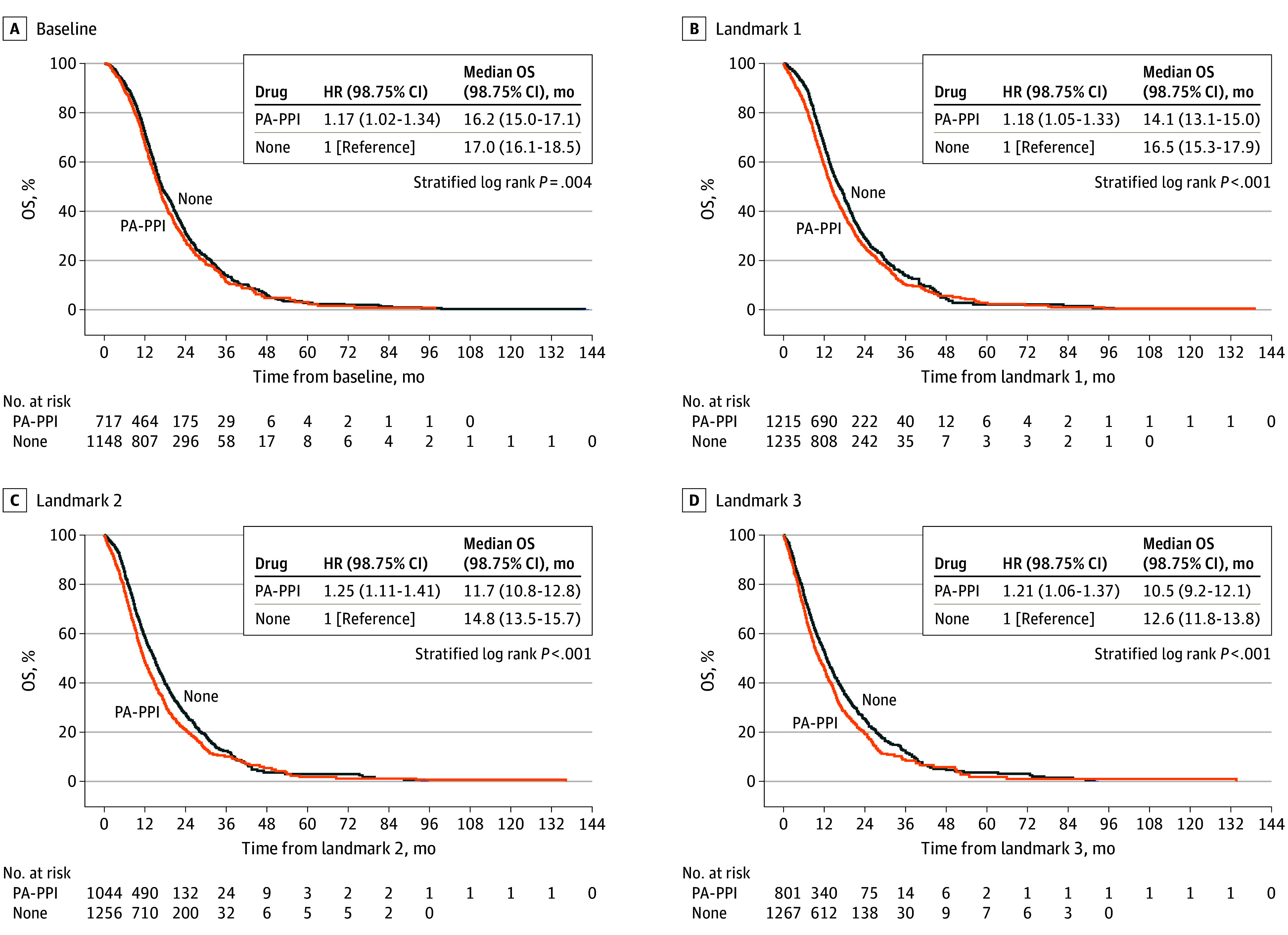
Associations of the Use of Potent ALDH1A1-Activating Proton Pump Inhibitors (PA-PPIs) With Overall Survival (OS) in Patients With Newly Diagnosed Glioblastoma HR indicates hazard ratio; landmark 1, start of maintenance cycle 1; landmark 2, start of maintenance cycle 4; landmark 3, end of maintenance cycle 6.

We first compared patients treated with PA-PPIs alone or in combination with other agents with patients who did not use any drug. On univariate analysis, we noted inferior PFS and OS for patients treated with PA-PPIs at all 4 time points (Figure 1; eFigure 2 in Supplement 1). Multivariate Cox models confirmed inferior PFS with the use of PA-PPIs at landmarks 1 (hazard ratio [HR], 1.14 [95% CI, 1.01-1.28]), 2 (HR, 1.26 [95% CI, 1.09-1.44]), and 3 (HR, 1.31 [95% CI, 1.10-1.56]) (Table 2). OS differences were significant at landmarks 1 (HR, 1.34 [95% CI, 1.08-1.66]) and 2 (HR, 1.14 [95% CI, 1.01-1.29]). Although univariate analysis indicated a statistically significant difference in OS at landmarks 1 and 2 for the use of other antacid drugs (eFigure 3 in Supplement 1), no such association was seen after adjusting for the other prognostic factors (Table 2).

**Table 2.** Adjusted Progression-Free and Overall Survival by PA-PPI or Antacid Use vs No Drug Intake

Drug categories	Survival					
	Progression-free			Overall		
	Events, No./total patients, No.	HR (98.75% CI)^a^	*P* value	Events, No./total patients, No.	HR (98.75% CI)^a^	*P* value
**PA-PPI with or without others**						
Baseline						
PA-PPI	660/713	1.07 (0.93-1.23)	.24	578/713	1.04 (0.90-1.21)	.47
Antacid	249/281	1.07 (0.88-1.30)	.38	216/281	1.02 (0.82-1.26)	.86
None	1038/1144	1 [Reference]	NA	912/1144	1 [Reference]	NA
Landmark 1^b^						
PA-PPI	954/1034	1.14 (1.01-1.28)	.009	974/1214	1.34 (1.08-1.66)	<.001
Antacid	294/334	1.03 (0.86-1.24)	.64	300/380	1.14 (0.95-1.37)	.07
None	975/1111	1 [Reference]	NA	917/1231	1 [Reference]	NA
Landmark 2^c^						
PA-PPI	653/720	1.26 (1.09-1.44)	<.001	841/1043	1.14 (1.01-1.29)	.009
Antacid	178/207	1.01 (0.81-1.25)	.96	237/304	1.11 (0.91-1.34)	.20
None	791/930	1 [Reference]	NA	930/1252	1 [Reference]	NA
Landmark 3^d^						
PA-PPI	383/440	1.31 (1.10-1.56)	<.001	628/801	1.13 (0.99-1.30)	.02^e^
Antacid	123/146	1.17 (0.90-1.51)	.14	174/233	1.08 (0.87-1.34)	.38^f^
None	570/719	1 [Reference]	NA	921/1263	1 [Reference]	NA

Abbreviations: ALDH1A1, aldehyde dehydrogenase 1 A1; HR, hazard ratio; NA, not applicable; PA-PPI, potent ALDH1A1 activating proton pump inhibitor (PPI).

^a^ Models were adjusted for baseline age, sex, *MGMT* promoter methylation status, extent of resection, most recent steroid use and World Health Organization performance status. All HRs and *P* values reported are from complete-case analysis.

^b^ Landmark 1 was defined as the start of maintenance cycle 1.

^c^ Landmark 2 was defined as the start of maintenance cycle 4.

^d^ Landmark 3 was defined as the end of maintenance cycle. Multiple imputation techniques were applied at landmark 3 overall survival analysis only due to 167 (6.8%) patients having missing drug information at this landmark.

^e^ Similar conclusion from multiple imputation: HR, 1.11 (98.75% CI, 0.97-1.27); *P* = .05.

^f^ Similar conclusion from multiple imputation: HR, 1.08 (98.75% CI, 0.87-1.33); *P* = .39.

### Outcomes Associated With PPI Use and *MGMT* Promoter Methylation Status

**Figure 2.**
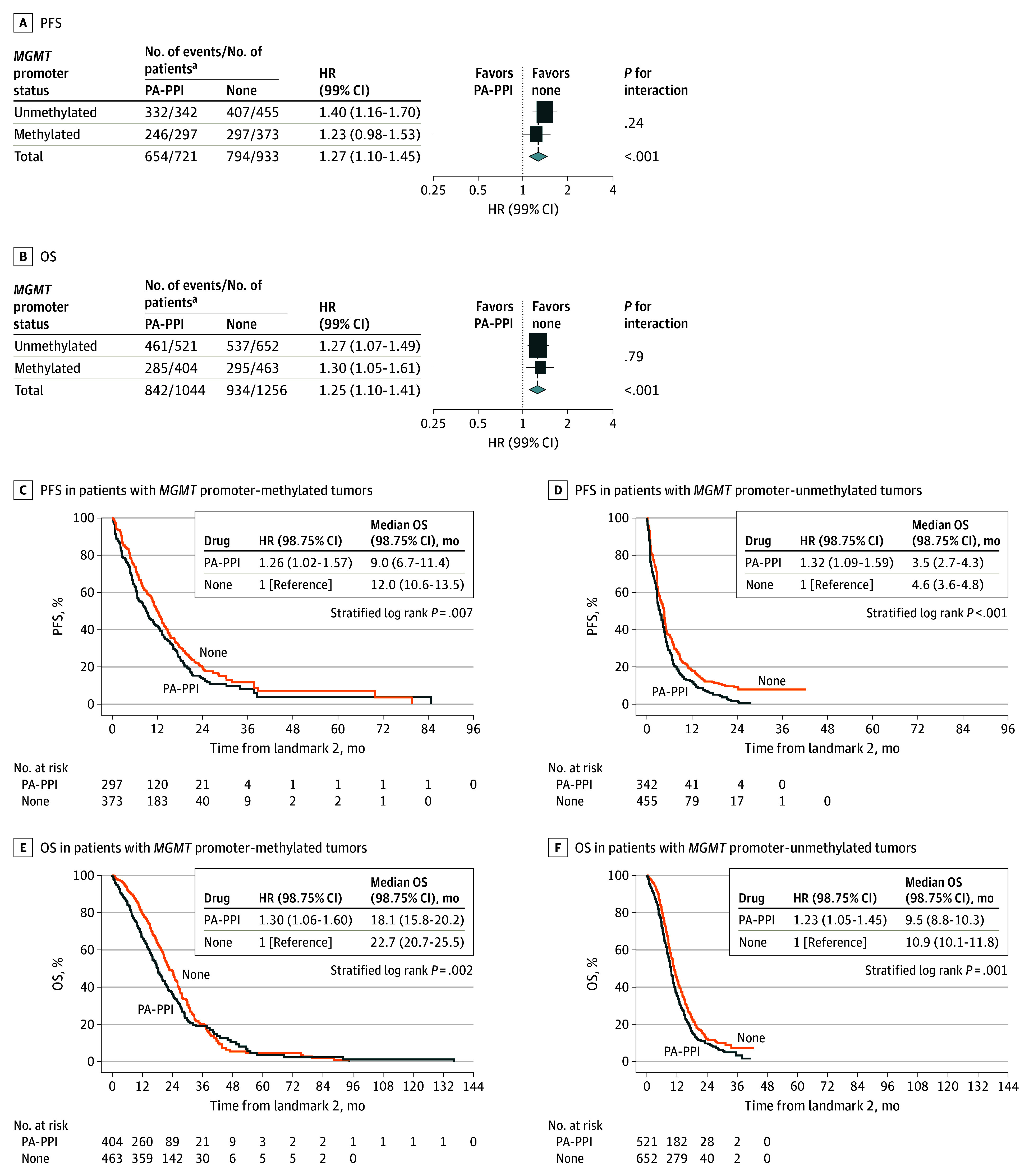
Associations of Potent ALDH1A1-Activating Proton Pump Inhibitors (PA-PPIs) Use With Outcome in Patients With Newly Diagnosed Glioblastoma Stratified by *MGMT* Promoter Methylation Status at Landmark 2 Landmark 2 was defined as the start of maintenance cycle 4. OS indicates overall survival; PFS, progression-free survival. Size of boxes (A and B) indicates statistical weight.

To explore whether PPI use may specifically counteract the antitumor effects of temozolomide, we assessed whether the inferior outcomes associated with PPI use were more prominent in patients with *MGMT* promoter-methylated tumors who are likely to derive greater benefit from temozolomide. However, interactions of PA-PPI use and *MGMT* status were not statistically significant at any of the time points (Figure 2; eFigure 4 in Supplement 1). The Kaplan-Meier plots revealed that the median survival for the patients with *MGMT* promoter-methylated tumors was longer than for the patients with unmethylated tumors; however, the forest plots indicated that for both subgroups, the association with PPI use remained detrimental compared with no drug. To confirm that the experimental treatments administered in the respective trials were not confounding factors, we performed the same analysis restricted to patients assigned to the control groups of these trials. We observed that there was no difference in PPI use by treatment group in these trials (eTables 6-10 in Supplement 1). The analysis of patients allocated to standard care found the same result as seen for the complete population, with similar magnitude of inferior outcome with PPI use independent of *MGMT* promoter methylation status (eFigure 4 and eFigure 5 in Supplement 1), although significance was lost with the reduced sample size.

### Interactions of PPI and Steroid Use

**Figure 3.**
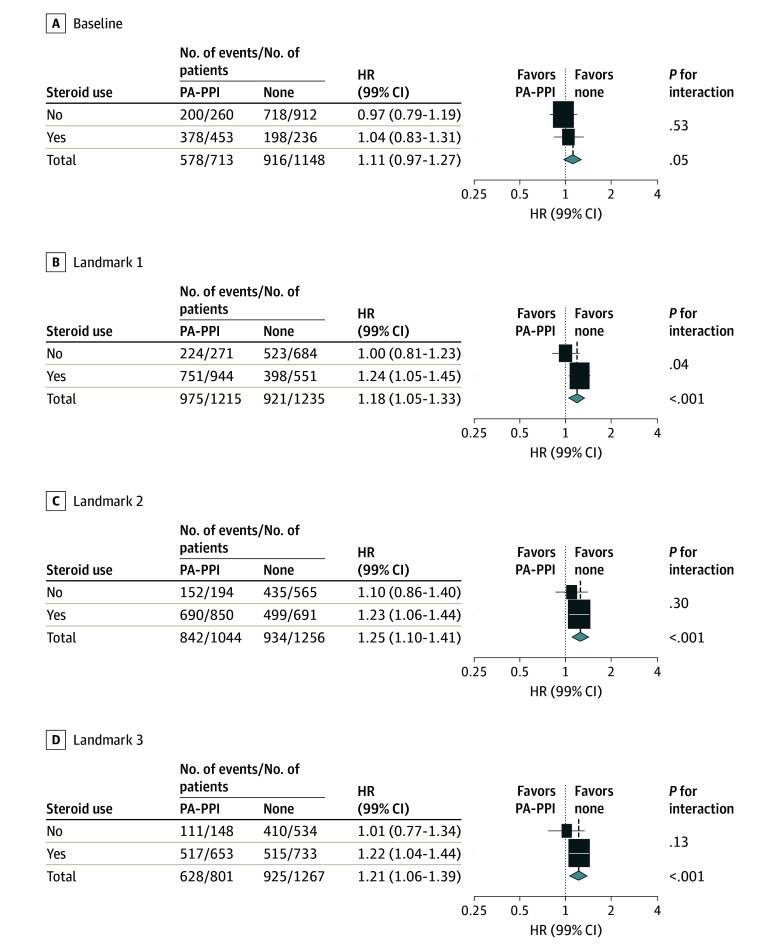
Interaction of Steroid Use and Potent ALDH1A1-Activating Proton Pump Inhibitors (PA-PPIs) Use for Overall Survival Associations HR indicates hazard ratio, landmark 1, start of maintenance cycle 1; landmark 2, start of maintenance cycle 4; landmark 3, end of maintenance cycle 6. Size of boxes indicates statistical weight.

Patients who were treated with steroids were more likely to be prescribed PPI or other antacid drugs than those who were not treated with steroids (eTable 11 in Supplement 1). To explore the association of steroid intake with the outcomes associated with PPI use, forest plots were obtained with interaction tests. None of the tests indicated statistical significance for the interaction of steroid and drug use. Although not statistically significant, the detrimental association of PPI use might be more prominent when steroids were administered (landmarks 1, 2, and 3) (Figure 3; eFigure 6 in Supplement 1), whereas no such trend was observed for the other, non-PPI antacid drugs and steroid use (eFigure 7 in Supplement 1).

## Discussion

The findings of this meta-analysis suggest that any outcomes associated with PA-PPI in patients with newly diagnosed glioblastoma are detrimental rather than beneficial. The in vitro findings of sensitization to chemotherapy by overcoming various pathways of resistance^3,4,5^ did not translate into the in vivo setting. Importantly, there was no association with outcome when antacid combinations other than PA-PPIs were used.

The precise mechanisms how PA-PPIs may affect outcomes, beyond a pure association, remain to be further studied. Blood-brain barrier permeability has been reported for some PPIs,^23,24^ raising the possibility that there are direct activities of PPIs on the tumors or their microenvironment. Intriguingly, a discussion on detrimental effects of PPIs on cognitive health has recently emerged, too,^25,26^ and linked to direct central nervous system effects of these drugs.

However, interactions can already occur at the level of drug absorption,^27,28^ eg, as recently suggested for palbociclib in patients with breast cancer.^29^ PPIs, by virtue of their action, also have profound effects on the microbiome, which in turn is relevant not only to the efficacy of immunotherapy, but potentially also natural immune responses to cancer.^30^ The most convincing hypothesis to explain the negative outcome associations in patients with glioblastoma reported here remains the activation of ALDH1A1 conferred by some PPIs (eAppendix 2 in Supplement 1).^9,11^

The analysis of associations with outcome over time revealed the strongest association during the landmarks of active treatment, suggesting that PPIs interfere with the efficacy of treatment. Yet, the outcome association was not restricted to patients with *MGMT* promoter-methylated glioblastoma, indicating that PPIs do not simply decrease the availability or efficacy of temozolomide. Since the previous analysis based on electronic medical records had reported a link of the negative outcomes of PPI use associated with *MGMT* promoter methylation, we performed a sensitivity analysis restricted to the patients enrolled into the control groups of the respective trials, but findings were essentially the same: *MGMT* promoter methylation status-independent negative outcomes were associated with PPI use. Importantly, patients with glioblastoma enrolled into clinical trials differ in many important prognostic factors from the general population with this disease, potentially explaining these discrepant results, and our dataset allowed us to control for important prognostic confounders, including age, extent of surgery, and performance status. Furthermore, although their 95% CIs crossed the null, the hazard ratios suggested a detriment already at baseline, prior to radiotherapy and temozolomide, which suggests that any cytoprotective mechanisms, eg, involving pH changes, are rapidly induced by PPIs and that these translate into inferior activity of the ensuing treatment with time.

We have previously reported a detrimental association of steroid use with outcomes in glioblastoma^15^ that may involve multiple mechanisms, including immunosuppression and tumor-intrinsic effects, eg, induction of resistance to radiotherapy or alkylating chemotherapy. Although PPI use was more common in patients treated with steroids, the same was true for other antacid drugs; moreover, PPI use remained prognostic in multivariate analysis that accounted for steroid use.

Most patients who were treated with PPIs may have been using primary prophylaxis, because no gastric complaints were documented. There are no specific guidelines for the prevention of gastric ulcers in patients exposed to steroids. PPIs are recommended for the prevention of ulcers in patients exposed to nonsteroidal anti-inflammatory agents for more than 3 months,^31^ even with no history of ulcers. Other recommendations argue against the routine prescription of PPIs in patients using steroids.^32^

Translational research studies may explore whether PPI-induced activity of ALDH1A1 mediates a potential detrimental effect of PPIs in glioblastoma or whether other pathways are involved. More importantly, no funding institution will engage in supporting a large phase 3 trial to prove that PPI exposure is detrimental, and why would patients consent to participation if the threat of inferior outcome is already communicated in the public domain and if alternatives to PPIs are available? In that sense, this analysis may substitute for a practice-changing trial for the years to come.

### Limitations

Our study has several limitations. This is a retrospective analysis, although data were captured prospectively. Drug use was only captured as long as the patients remained in the trial, and there may be confounding effects on the OS analyses by pharmacotherapy continued or started after progression, when patients discontinued study follow-up. Patients with more aggressive tumors or more symptomatic edema are likely to have both higher steroid doses and more frequent PPI use, creating a confounding cluster of factors associated with worse prognosis. Yet, the associations with PFS remain unaffected by this limitation. Overall steroid dose, duration of treatment, or timing relative to treatment phases were not captured, suggesting that the independence of negative outcomes of PPIs associated with steroid use should not be overstated. Furthermore, there may be underreporting of drug intake, notably of over-the-counter medications.

## Conclusions

In this meta-analysis of patients with newly diagnosed glioblastoma, PPI use was associated with inferior outcomes. These findings suggest that routine prophylactic PPI use should be discouraged in patients with glioblastoma, since alternative agents, such as H2 blockers or locally acting antacids, are available and since a detrimental effect cannot be excluded.

## Notes

Supplement 1. eTable 1. Clinical trials included in this analysis eTable 2. Classification of relevant comedications eTable 3. Patient characteristics at baseline and outcome eTable 4. Frequency of use of drugs of interest eTable 5. Correlation between documentation of gastric complaints and use of drugs of interest eTable 6. Drug use by trial group in the ACT-IV trial eTable 7. Drug use by trial group in the AVAglio trial eTable 8. Drug use by trial group in the EORTC 26071 (CENTRIC) trial eTable 9. Drug use by trial group in the CORE trial eTable 10. Drug use by trial group in the EORTC 1709 (MIRAGE) trial eTable 11. Correlation between use of steroids and use of drugs of interest eFigure 1. CONSORT diagram eFigure 2. Associations of the use of PA-PPI with progression-free survival outcome in patients with newly diagnosed glioblastoma eFigure 3. Associations of AA with or without weak ALDH1A1-activating PPI use with outcome in patients with newly diagnosed glioblastoma eFigure 4. Associations of PA-PPI use with outcome in patients with newly diagnosed glioblastoma stratified by *MGMT* promoter methylation status at landmark 1 eFigure 5. Associations of PA-PPI use with outcome in patients with newly diagnosed glioblastoma assigned to the control groups of the five trials, stratified by *MGMT* promoter methylation status at landmark 2 eFigure 6. Interaction of steroid and PA-PPI use for progression-free survival associations eFigure 7. Interaction of steroid and AA (without PA-PPI) use for outcome associations eAppendix 1. Details of multiple imputation eAppendix 2. PPI and ALDH1 eReferences.

Supplement 2. Data Sharing Statement
