# Supplementary material for: Proton Pump Inhibitor Use and Survival in Patients With Newly Diagnosed Glioblastoma
Source: JAMA Netw Open. 2025 Nov 25;8(11):e2545578. doi: 10.1001/jamanetworkopen.2025.45578 (PMC12648345; doi:10.1001/jamanetworkopen.2025.45578)
Supplement: Supplement 1. — eTable 1. Clinical trials included in this analysis eTable 2. Classification of relevant comedications eTable 3. Patient characteristics at baseline and outcome eTable 4. Frequency of use of drugs of interest eTable 5. Correlation between documentation of gastric complaints and use of drugs of interest eTable 6. Drug use by trial group in the ACT-IV trial eTable 7. Drug use by trial group in the AVAglio trial eTable 8. Drug use by trial group in the EORTC 26071 (CENTRIC) trial eTable 9. Drug use by trial group in the CORE trial eTable 10. Drug use by trial group in the EORTC 1709 (MIRAGE) trial eTable 11. Correlation between use of steroids and use of drugs of interest eFigure 1. CONSORT diagram eFigure 2. Associations of the use of PA-PPI with progression-free survival outcome in patients with newly diagnosed glioblastoma eFigure 3. Associations of AA with or without weak ALDH1A1-activating PPI use with outcome in patients with newly diagnosed glioblastoma eFigure 4. Associations of PA-PPI use with outcome in patients with newly diagnosed glioblastoma stratified by MGMT promoter methylation status at landmark 1 eFigure 5. Associations of PA-PPI use with outcome in patients with newly diagnosed glioblastoma assigned to the control groups of the five trials, stratified by MGMT promoter methylation status at landmark 2 eFigure 6. Interaction of steroid and PA-PPI use for progression-free survival associations eFigure 7. Interaction of steroid and AA (without PA-PPI) use for outcome associations eAppendix 1. Details of multiple imputation eAppendix 2. PPI and ALDH1 eReferences. [file jamanetwopen-e2545578-s001.pdf]

# Supplemental Online Content

Le Rhun E, Sain D, Erridge SC, et al. Proton pump inhibitor use and survival in patients with newly diagnosed glioblastoma. *JAMA Netw Open*. 2025;8(11):e2545578. doi:10.1001/jamanetworkopen.2025.45578

eTable 1. Clinical trials included in this analysis
eTable 2. Classification of relevant comedications
eTable 3. Patient characteristics at baseline and outcome
eTable 4. Frequency of use of drugs of interest
eTable 5. Correlation between documentation of gastric complaints and use of drugs of interest
eTable 6. Drug use by trial group in the ACT-IV trial
eTable 7. Drug use by trial group in the AVAglio trial
eTable 8. Drug use by trial group in the EORTC 26071 (CENTRIC) trial
eTable 9. Drug use by trial group in the CORE trial
eTable 10. Drug use by trial group in the EORTC 1709 (MIRAGE) trial
eTable 11. Correlation between use of steroids and use of drugs of interest
eFigure 1. CONSORT diagram
eFigure 2. Associations of the use of PA-PPI with progression-free survival outcome in patients with newly diagnosed glioblastoma
eFigure 3. Associations of AA with or without weak ALDH1A1-activating PPI use with outcome in patients with newly diagnosed glioblastoma
eFigure 4. Associations of PA-PPI use with outcome in patients with newly diagnosed glioblastoma stratified by MGMT promoter methylation status at landmark 1
eFigure 5. Associations of PA-PPI use with outcome in patients with newly diagnosed glioblastoma assigned to the control groups of the five trials, stratified by MGMT promoter methylation status at landmark 2
eFigure 6. Interaction of steroid and PA-PPI use for progression-free survival associations
eFigure 7. Interaction of steroid and AA (without PA-PPI) use for outcome associations
eAppendix 1. Details of multiple imputation
eAppendix 2. PPI and ALDH1
eReferences.

This supplemental material has been provided by the authors to give readers additional information about their work.

© 2025 Le Rhun E et al. *JAMA Network Open*.

**eTable 1. Clinical trials included in this analysis.**

| Trial | NCT number | Title | Primary report (PMID) |
|---|---|---|---|
| | | | |
| ACT IV | NCT01480479 | Phase III Study of Rindopepimut/GM-CSF in Patients With Newly Diagnosed Glioblastoma | PMID 28844499 (1) |
| | | | |
| AVAglio | NCT00943826 | A Study of Bevacizumab (Avastin®) in Combination With Temozolomide and Radiotherapy in Participants With Newly Diagnosed Glioblastoma | PMID 24552318 (2) |
| | | | |
| EORTC 26071 CENTRIC | NCT00689221 | Cilengitide, Temozolomide, and Radiation Therapy in Treating Patients With Newly Diagnosed Glioblastoma and Methylated Gene Promoter Status | PMID 15758009 (3) |
| | | | |
| CORE | NCT00085254 | Cilengitide, Temozolomide, and Radiation Therapy in Treating Patients With Newly Diagnosed Glioblastoma Multiforme | PMID 25762461 (4) |
| | | | |
| EORTC 1709 | NCT03345095 | A Phase III Trial of With Marizomib in Patients With Newly Diagnosed Glioblastoma | PMID 38502052 (5) |
| | | | |

Patients with known isocitrate dehydrogenase 1 or 2 mutation or 1p/19q codeletion were excluded from this analysis. All analyses were stratified by trial, in addition, only for AVAglio, stratification included consideration of treatment group because of improved PFS in the experimental arm.

© 2025 Le Rhun E et al. *JAMA Network Open*.

**eTable 2. Classification of relevant comedications (Calleja et al., 2020)** (6)

| Class | Drugs |
|---|---|
| | |
| PPI with potent ALDH1A1 activation (PA PPI) | Omeprazole (esomeprazole by extrapolation), pantoprazole |
| | |
| PPI with weak ALDH1A1 activation | Lansoprazole (dexlansoprazole), rabeprazole |
| | |
| H2-blocking AA | Cimetidine, famotidine, nizatidine, ranitidine, roxatidine, lafutidine |
| | |
| Locally acting AA | Aluminum/magnesium antacids such as Maalox, Gaviscon, and many others etc. |

Abbreviations: AA, anti-acids; ALDH1A1, aldehyde dehydrogenase 1 A1 (ALDH1A1); PA-PPI, potent ALDH1A1 activating PPI; PPI proton pump inhibitor.

© 2025 Le Rhun E et al. *JAMA Network Open*.

**eTable 3. Patient characteristics at baseline and outcome.**

| | Trial | | | | | Total N=2981 |
|---|---|---|---|---|---|---|
| | ACT-IV N=745 | AVAglio N=921 | CENTRIC N=379 | CORE N=197 | EORTC 1709 N=739 | |
| | N (%) | N (%) | N (%) | N (%) | N (%) | N (%) |
| **Age** | | | | | | |
| <50 years | 152 (20.4) | 229 (24.9) | 67 (17.7) | 51 (25.9) | 167 (22.6) | 666 (22.3) |
| 50-64 years | 419 (56.2) | 492 (53.4) | 207 (54.6) | 112 (56.9) | 387 (52.4) | 1617 (54.2) |
| ≥65years | 174 (23.4) | 200 (21.7) | 105 (27.7) | 34 (17.3) | 185 (25.0) | 698 (23.4) |
| **Sex** | | | | | | |
| Male | 480 (64.4) | 580 (63.0) | 200 (52.8) | 118 (59.9) | 480 (65.0) | 1858 (62.3) |
| Female | 265 (35.6) | 341 (37.0) | 179 (47.2) | 79 (40.1) | 259 (35.0) | 1123 (37.7) |
| **Steroid use** | | | | | | |
| No | 387 (51.9) | 522 (56.7) | 219 (57.8) | 120 (60.9) | 432 (58.5) | 1680 (56.4) |
| Yes | 358 (48.1) | 395 (42.9) | 160 (42.2) | 77 (39.1) | 307 (41.5) | 1297 (43.5) |
| Missing | 0 (0.0) | 4 (0.4) | 0 (0.0) | 0 (0.0) | 0 (0.0) | 4 (0.1) |
| ***MGMT* promoter status** | | | | | | |
| Unmethylated | 439 (58.9) | 462 (50.2) | 0 (0.0) | 197 (100.0) | 434 (58.7) | 1532 (51.4) |
| Methylated | 253 (34.0) | 237 (25.7) | 379 (100.0) | 0 (0.0) | 234 (31.7) | 1103 (37.0) |
| Unknown | 53 (7.1) | 222 (24.1) | 0 (0.0) | 0 (0.0) | 71 (9.6) | 346 (11.6) |
| **WHO performance status** | | | | | | |
| 0 | 339 (45.5) | 630 (68.4) | 219 (57.8) | 96 (48.7) | 163 (22.1) | 1447 (48.5) |
| >0 | 402 (54.0) | 289 (31.4) | 160 (42.2) | 101 (51.3) | 576 (77.9) | 1528 (51.3) |
| Missing | 4 (0.5) | 2 (0.2) | 0 (0.0) | 0 (0.0) | 0 (0.0) | 6 (0.2) |
| **Extent of surgery** | | | | | | |
| Partial resection or biopsy | 280 (37.6) | 537 (58.3) | 171 (45.1) | 93 (47.2) | 368 (49.8) | 1449 (48.6) |
| Gross total resection | 465 (62.4) | 384 (41.7) | 206 (54.4) | 103 (52.3) | 371 (50.2) | 1529 (51.3) |
| Missing | 0 (0.0) | 0 (0.0) | 2 (0.5) | 1 (0.5) | 0 (0.0) | 3 (0.1) |
| **Median progression-free survival (months)** | | | | | | |
| **Standard arm** | 5.6 | 6.2 | 10.7 | 4.1 | 6.0 | n.a. |
| **Experimental arm** | 7.1 | 10.6 | 13.5 | 5.6/5.9 | 6.3 | n.a. |
| **Median overall survival (months)** | | | | | | |
| **Standard arm** | 20.0 | 16.7 | 26.3 | 13.4 | 17.0 | n.a. |
| **Experimental arm** | 20.1 | 16.8 | 26.3 | 16.3/14.5 | 16.5 | n.a. |

As ACT-IV randomized patients after the concomitant chemoradiotherapy phase and did not have a baseline like other trials, patient characteristics at randomization is reported; CORE had two experimental arms, low and high dose cilengitide (n.a., not applicable).

Abbreviations: MGMT, $O^6$-methylguanine DNA methyltransferase; WHO World Health Organization.

© 2025 Le Rhun E et al. *JAMA Network Open*.

## eTable 4. Frequency of use of drugs of interest.

| Drug Categories (at all times) for patients with newly diagnosed glioblastoma | | | | | | |
|---|---|---|---|---|---|---|
| | Trial | | | | | Total (N=2981) |
| | ACT-IV (N=745) | AVAglio (N=921) | CENTRIC (N=379) | CORE (N=197) | EORTC 1709 (N=739) | |
| **Drug categories** | **N (%)** | **N (%)** | **N (%)** | **N (%)** | **N (%)** | **N (%)** |
| **None** | 292 (39.2) | 150 (16.3) | 90 (23.7) | 39 (19.8) | 255 (34.5) | 826 (27.7) |
| **AA only** | 120 (16.1) | 147 (16.0) | 51 (13.5) | 41 (20.8) | 56 (7.6) | 415 (13.9) |
| **Weak ALDH1A1-activating PPI only** | 15 (2.0) | 48 (5.2) | 11 (2.9) | 13 (6.6) | 37 (5.0) | 124 (4.2) |
| **Weak ALDHA1-activating PPI plus AA** | 4 (0.5) | 14 (1.5) | 1 (0.3) | 2 (1.0) | 9 (1.2) | 30 (1.0) |
| **PA-PPI only** | 250 (33.6) | 406 (44.1) | 176 (46.4) | 83 (42.1) | 352 (47.6) | 1267 (42.5) |
| **PA-PPI plus AA** | 57 (7.7) | 114 (12.4) | 39 (10.3) | 16 (8.1) | 23 (3.1) | 249 (8.4) |
| **PA-PPI plus weak ALDH1A1-activating PPI** | 5 (0.7) | 32 (3.5) | 7 (1.8) | 2 (1.0) | 5 (0.7) | 51 (1.7) |
| **PA-PPI plus weak ALDH1A1-activating PPI plus anti-acids** | 2 (0.3) | 10 (1.1) | 4 (1.1) | 1 (0.5) | 2 (0.3) | 19 (0.6) |

The table shows the cumulative patterns of drug administrations over the entire observation period on a patient level for all 2981 patients. For further analysis, we regrouped drug categories as follows: “All PA PPI” as PA-PPI only, PA-PPI plus anti-acids, PA-PPI plus weak ALDH1A1-activating PPI, and PA-PPI plus weak ALDH1-activating PPI plus anti-acids; “All AA without PA-PPI” as anti-acid only alone or in combination with weak ALDH1A1-activating PPI, and “none” for patients who did not use any drug (see also Table S2).

Abbreviations: AA, anti-acids; ALDH1A1, aldehyde dehydrogenase 1 A1 (ALDH1A1); PA-PPI, potent ALDH1A1 activating PPI; PPI proton pump inhibitor.

© 2025 Le Rhun E et al. *JAMA Network Open*.

**eTable 5. Correlation between documentation of gastric complaints and use of drugs of interest.**

| | None | | All PA-PPI combinations | | All other AA combinations | |
|---|---|---|---|---|---|---|
| Gastric complaints, n (%) | no | yes | no | yes | No | yes |
| | | | | | | |
| Baseline | 1128 (98.3) | 20 (1.7) | 649 (90.5) | 68 (9.5) | 260 (92.2) | 22 (7.8) |
| | | | | | | |
| Progression-free survival | | | | | | |
| | | | | | | |
| Landmark 1 | 1099 (98.7) | 15 (1.3) | 944 (91.2) | 91 (8.8) | 313 (93.7) | 21 (6.3) |
| | | | | | | |
| Landmark 2 | 906 (97.1) | 27 (2.9) | 650 (90.3) | 70 (9.7) | 188 (90.8) | 19 (9.2) |
| | | | | | | |
| Landmark 3 | 706 (97.8) | 16 (2.2) | 404 (92.0) | 35 (8.0) | 129 (87.8) | 18 (12.2) |
| | | | | | | |
| Overall survival | | | | | | |
| | | | | | | |
| Landmark 1 | 1219 (98.7) | 16 (1.3) | 1106 (91.0) | 109 (9.0) | 355 (93.4) | 25 (6.6) |
| | | | | | | |
| Landmark 2 | 1224 (97.5) | 32 (2.5) | 954 (91.5) | 89 (8.5) | 278 (91.4) | 26 (8.6) |
| | | | | | | |
| Landmark 3 | 1243 (98.3) | 22 (1.7) | 740 (92.5) | 60 (7.5) | 213 (91.0) | 21 (9.0) |

Abbreviations: AA, anti-acids; ALDH1A1, aldehyde dehydrogenase 1 A1 (ALDH1A1); PA-PPI, potent ALDH1A1 activating PPI; PPI proton pump inhibitor.

© 2025 Le Rhun E et al. *JAMA Network Open*.

**eTable 6. Drug use by trial group in the ACT-IV trial.**

| | Patient numbers - N (%) | | |
|---|---|---|---|
| **Landmark 1, progression-free survival** | **Placebo+TMZ** 359 (100) | **Rindopepimut+TMZ** 358 (100) | **Total** 717 (100) |
| …..None | 202 (56.3) | 195 (54.5) | 397 (55.4) |
| All PA-PPI combinations | 109 (30.4) | 113 (31.6) | 222 (31.0) |
| All other AA combinations | 48 (13.4) | 50 (14.0) | 98 (13.7) |
| | | | |
| **Landmark 2, progression-free survival** | **Placebo +TMZ** 266 (100) | **Rindopepimut+TMZ** 282 (100) | **Total** 548 (100) |
| …..None | 139 (52.3) | 149 (52.8) | 288 (52.6) |
| All PA-PPI combinations | 96 (36.1) | 92 (32.6) | 188 (34.3) |
| All other AA combinations | 31 (11.7) | 41 (14.5) | 72 (13.1) |
| | | | |
| **Landmark 3, progression-free survival** | **Placebo+TMZ** 195 (100) | **Rindopepimut+TMZ** 205 (100) | **Total** 400 (100) |
| …..None | 108 (55.4) | 103 (50.2) | 211 (52.8) |
| All PA-PPI combinations | 60 (30.8) | 70 (34.1) | 130 (32.5) |
| All other AA combinations | 27 (13.8) | 32 (15.6) | 59 (14.8) |
| | | | |
| **Landmark 1, overall survival** | **Placebo+TMZ** 364 (100) | **Rindopepimut+TMZ** 363 (100) | **Total** 727 (100) |
| …..None | 204 (56.0) | 198 (54.5) | 402 (55.3) |
| All PA-PPI combinations | 112 (30.8) | 114 (31.4) | 226 (31.1) |
| All other AA combinations | 48 (13.2) | 51 (14.0) | 99 (13.6) |
| | | | |
| **Landmark 2, overall survival** | **Placebo+TMZ** 353 (100) | **Rindopepimut+TMZ** 354 (100) | **Total** 707 (100) |
| …..None | 184 (52.1) | 184 (52.0) | 368 (52.1) |
| All PA-PPI combinations | 127 (36.0) | 119 (33.6) | 246 (34.8) |
| All other AA combinations | 42 (11.9) | 51 (14.4) | 93 (13.2) |
| | | | |
| **Landmark 3, overall survival** | **Placebo+TMZ** 336 (100) | **Rindopepimut+TMZ** 339 (100) | **Total** 675 (100) |
| …..None | 186 (55.4) | 180 (53.1) | 366 (54.2) |
| All PA-PPI combinations | 111 (33.0) | 110 (32.4) | 221 (32.7) |
| All other AA combinations | 39 (11.6) | 49 (14.5) | 88 (13.0) |

Abbreviations: AA, anti-acids; ALDH1A1, aldehyde dehydrogenase 1 A1 (ALDH1A1); PA-PPI, potent ALDH1A1 activating PPI; PPI proton pump inhibitor; TMZ, temozolomide.

© 2025 Le Rhun E et al. *JAMA Network Open*.

**eTable 7. Drug use by trial group in the AVAglio trial.**

| | Patient numbers - N (%) | | |
|---|---|---|---|
| **Baseline, progression-free and overall survival** | **Placebo+TMZ+RT** 449 (100) | **Bevacizumab+TMZ+RT** 431 (100) | **Total** 880 (100) |
| …..None | 180 (40.1) | 196 (45.5) | 376 (42.7) |
| All PA-PPI combinations | 178 (39.6) | 166 (38.5) | 344 (39.1) |
| All other AA combinations | 91 (20.3) | 69 (16.0) | 160 (18.2) |
| | | | |
| **Landmark 1, progression-free survival** | **Placebo+TMZ+RT** 355 (100) | **Bevacizumab+TMZ+RT** 416 (100) | **Total** 771 (100) |
| …..None | 129 (36.3) | 160 (38.5) | 289 (37.5) |
| All PA-PPI combinations | 167 (47.0) | 196 (47.1) | 363 (47.1) |
| All other AA combinations | 59 (16.6) | 60 (14.4) | 119 (15.4) |
| | | | |
| **Landmark 2, progression-free survival** | **Placebo+TMZ+RT** 255 (100) | **Bevacizumab+TMZ+RT** 368 (100) | **Total** 623 (100) |
| …..None | 117 (45.9) | 189 (51.4) | 306 (49.1) |
| All PA-PPI combinations | 107 (42.0) | 136 (37.0) | 243 (39.0) |
| All other AA combinations | 31 (12.2) | 43 (11.7) | 74 (11.9) |
| | | | |
| **Landmark 3, progression-free survival** | **Placebo+TMZ+RT** 165 (100) | **Bevacizumab+TMZ+RT** 287 (100) | **Total** 452 (100) |
| …..None | 93 (56.4) | 168 (58.5) | 261 (57.7) |
| All PA-PPI combinations | 52 (31.5) | 91 (31.7) | 143 (31.6) |
| All other AA combinations | 20 (12.1) | 28 (9.8) | 48 (10.6) |
| | | | |
| **Landmark 1, overall survival** | **Placebo+TMZ+RT** 434 (100) | **Bevacizumab+TMZ+RT** 429 (100) | **Total** 863 (100) |
| …..None | 151 (34.8) | 162 (37.8) | 313 (36.3) |
| All PA-PPI combinations | 210 (48.4) | 204 (47.6) | 414 (48.0) |
| All other AA combinations | 73 (16.8) | 63 (14.7) | 136 (15.8) |
| | | | |
| **Landmark 2, overall survival** | **Placebo+TMZ+RT** 388 (100) | **Bevacizumab+TMZ+RT** 401 (100) | **Total** 789 (100) |
| …..None | 169 (43.6) | 201 (50.1) | 370 (46.9) |
| All PA-PPI combinations | 165 (42.5) | 154 (38.4) | 319 (40.4) |
| All other AA combinations | 54 (13.9) | 45 (11.2) | 99 (12.5) |
| Not collected | 0 (0.0) | 1 (0.2) | 1 (0.1) |
| | | | |
| **Landmark 3, overall survival** | **Placebo+TMZ+RT** 365 (100) | **Bevacizumab+TMZ+RT** 384 (100) | **Total** 749 (100) |
| …..None | 173 (47.4) | 213 (55.5) | 386 (51.5) |
| All PA-PPI combinations | 126 (34.5) | 126 (32.8) | 252 (33.6) |
| All other AA combinations | 42 (11.5) | 39 (10.2) | 81 (10.8) |
| Not collected | 24 (6.6) | 6 (1.6) | 30 (4.0) |

Abbreviations: AA, anti-acids; ALDH1A1, aldehyde dehydrogenase 1 A1 (ALDH1A1); PA-PPI, potent ALDH1A1 activating PPI; PFS, PPI proton pump inhibitor; RT, radiotherapy, TMZ, temozolomide.

© 2025 Le Rhun E et al. *JAMA Network Open*.

**eTable 8. Drug use by trial group in the EORTC 26071 (CENTRIC) trial.**

| | Patient numbers - N (%) | | |
|---|---|---|---|
| **Baseline, progression-free and overall survival** | **Control** 188 (100) | **Cilengitide** 183 (100) | **Total** 371 (100) |
| …..None | 116 (61.7) | 106 (57.9) | 222 (59.8) |
| All PA-PPI combinations | 54 (28.7) | 57 (31.1) | 111 (29.9) |
| All other AA combinations | 18 (9.6) | 20 (10.9) | 38 (10.2) |
| | | | |
| **Landmark 1, progression-free survival** | **Control** 158 (100) | **Cilengitide** 156 (100) | **Total** 314 (100) |
| …..None | 63 (39.9) | 56 (35.9) | 119 (37.9) |
| All PA-PPI combinations | 77 (48.7) | 81 (51.9) | 158 (50.3) |
| All other AA combinations | 18 (11.4) | 19 (12.2) | 37 (11.8) |
| | | | |
| **Landmark 2, progression-free survival** | **Control** 113 (100) | **Cilengitide** 117 (100) | **Total** 230 (100) |
| …..None | 56 (49.6) | 50 (42.7) | 106 (46.1) |
| All PA-PPI combinations | 46 (40.7) | 53 (45.3) | 99 (43.0) |
| All other AA combinations | 11 (9.7) | 14 (12.0) | 25 (10.9) |
| | | | |
| **Landmark 3, progression-free survival** | **Control** 88 (100) | **Cilengitide** 97 (100) | **Total** 185 (100) |
| …..None | 57 (64.8) | 47 (48.5) | 104 (56.2) |
| All PA-PPI combinations | 27 (30.7) | 38 (39.2) | 65 (35.1) |
| All other AA combinations | 4 (4.5) | 12 (12.4) | 16 (8.6) |
| | | | |
| **Landmark 1, overall survival** | **Control** 184 (100) | **Cilengitide** 180 (100) | **Total** 364 (100) |
| …..None | 70 (38.0) | 65 (36.1) | 135 (37.1) |
| All PA-PPI combinations | 91 (49.5) | 93 (51.7) | 184 (50.5) |
| All other AA combinations | 23 (12.5) | 22 (12.2) | 45 (12.4) |
| | | | |
| **Landmark 2, overall survival** | **Control** 170 (100) | **Cilengitide** 160 (100) | **Total** 330 (100) |
| …..None | 78 (45.9) | 63 (39.4) | 141 (42.7) |
| All PA-PPI combinations | 73 (42.9) | 79 (49.4) | 152 (46.1) |
| All other AA combinations | 18 (10.6) | 17 (10.6) | 35 (10.6) |
| Not collected | 1 (0.6) | 1 (0.6) | 2 (0.6) |
| | | | |
| **Landmark 3, overall survival** | **Control** 150 (100) | **Cilengitide** 153 (100) | **Total** 303 (100) |
| …..None | 85 (56.7) | 71 (46.4) | 156 (51.5) |
| All PA-PPI combinations | 44 (29.3) | 59 (38.6) | 103 (34.0) |
| All other AA combinations | 7 (4.7) | 12 (7.8) | 19 (6.3) |
| Not collected | 14 (9.3) | 11 (7.2) | 25 (8.3) |

Abbreviations: AA, anti-acids; ALDH1A1, aldehyde dehydrogenase 1 A1 (ALDH1A1); PA-PPI, potent ALDH1A1 activating PPI; PPI proton pump inhibitor.

© 2025 Le Rhun E et al. *JAMA Network Open*.

**eTable 9. Drug use by trial group in the CORE trial.**

| | Patient numbers - N (%) | | | |
|---|---|---|---|---|
| **Baseline, progression-free and overall survival** | **Standard Cilengitide** 61 (100) | **Intensive Cilentigide** 64 (100) | **Control** 64 (100) | **Total** 189 (100) |
| …..None | 40 (65.6) | 44 (68.8) | 46 (71.9) | 130 (68.8) |
| All PA-PPI combinations | 16 (26.2) | 11 (17.2) | 9 (14.1) | 36 (19.0) |
| All other AA combinations | 5 (8.2) | 9 (14.1) | 9 (14.1) | 23 (12.2) |
| | | | | |
| **Landmark 1, progression-free survival** | **Standard Cilengitide** 49 (100) | **Intensive Cilentigide** 55 (100) | **Control** 48 (100) | **Total** 152 (100) |
| …..None | 21 (42.9) | 15 (27.3) | 21 (43.8) | 57 (37.5) |
| All PA-PPI combinations | 19 (38.8) | 25 (45.5) | 16 (33.3) | 60 (39.5) |
| All other AA combinations | 9 (18.4) | 15 (27.3) | 11 (22.9) | 35 (23.0) |
| | | | | |
| **Landmark 2, progression-free survival** | **Standard Cilengitide** 27 (100) | **Intensive Cilentigide** 39 (100) | **Control** 28 (100) | **Total** 94 (100) |
| …..None | 12 (44.4) | 15 (38.5) | 13 (46.4) | 40 (42.6) |
| All PA-PPI combinations | 11 (40.7) | 16 (41.0) | 11 (39.3) | 38 (40.4) |
| All other AA combinations | 4 (14.8) | 8 (20.5) | 4 (14.3) | 16 (17.0) |
| | | | | |
| **Landmark 3, progression-free survival** | **Standard Cilengitide** 13 (100) | **Intensive Cilentigide** 20 (100) | **Control** 7 (100) | **Total** 40 (100) |
| …..None | 7 (53.8) | 8 (40.0) | 4 (57.1) | 19 (47.5) |
| All PA-PPI combinations | 3 (23.1) | 8 (40.0) | 2 (28.6) | 13 (32.5) |
| All other AA combinations | 3 (23.1) | 4 (20.0) | 1 (14.3) | 8 (20.0) |
| | | | | |
| **Landmark 1, overall survival** | **Standard Cilengitide** 59 (100) | **Intensive Cilentigide** 61 (100) | **Control** 61 (100) | **Total** 181 (100) |
| …..None | 22 (37.3) | 18 (29.5) | 27 (44.3) | 67 (37.0) |
| All PA-PPI combinations | 26 (44.1) | 27 (44.3) | 21 (34.4) | 74 (40.9) |
| All other AA combinations | 11 (18.6) | 16 (26.2) | 13 (21.3) | 40 (22.1) |
| | | | | |
| **Landmark 2, overall survival** | **Standard Cilengitide** 54 (100) | **Intensive Cilentigide** 57 (100) | **Control** 53 (100) | **Total** 164 (100) |
| …..None | 21 (38.9) | 23 (40.4) | 23 (43.4) | 67 (40.9) |
| All PA-PPI combinations | 23 (42.6) | 22 (38.6) | 20 (37.7) | 65 (39.6) |
| All other AA combinations | 8 (14.8) | 12 (21.1) | 10 (18.9) | 30 (18.3) |
| Not collected | 2 (3.7) | 0 (0.0) | 0 (0.0) | 2 (1.2) |
| | | | | |
| **Landmark 3, overall survival** | **Standard Cilengitide** 45 (100) | **Intensive Cilentigide** 52 (100) | **Control** 48 (100) | **Total** 145 (100) |
| …..None | 17 (37.8) | 21 (40.4) | 23 (47.9) | 61 (42.1) |
| All PA-PPI combinations | 15 (33.3) | 18 (34.6) | 14 (29.2) | 47 (32.4) |
| All other AA combinations | 5 (11.1) | 8 (15.4) | 6 (12.5) | 19 (13.1) |
| Not collected | 8 (17.8) | 5 (9.6) | 5 (10.4) | 18 (12.4) |

Abbreviations: AA, anti-acids; ALDH1A1, aldehyde dehydrogenase 1 A1 (ALDH1A1); PA-PPI, potent ALDH1A1 activating PPI; PPI proton pump inhibitor.

© 2025 Le Rhun E et al. *JAMA Network Open*.

**eTable 10. Drug use by trial group in the EORTC 1709 (MIRAGE) trial.**

| | Patient numbers - N (%)survival | | |
|---|---|---|---|
| **Baseline, progression-free and overall** | **TMZ+RT**<br>349 (100) | **TMZ+RT+marizomib**<br>358 (100) | **Total**<br>707 (100) |
| …..None | 204 (58.5) | 216 (60.3) | 420 (59.4) |
| All PA-PPI combinations | 115 (33.0) | 111 (31.0) | 226 (32.0) |
| All other AA combinations | 30 (8.6) | 31 (8.7) | 61 (8.6) |
| | | | |
| **Landmark 1, progression-free survival** | **TMZ+RT**<br>253 (100) | **TMZ+RT+ marizomib**<br>276 (100) | **Total**<br>529 (100) |
| …..None | 112 (44.3) | 140 (50.7) | 252 (47.6) |
| All PA-PPI combinations | 117 (46.2) | 115 (41.7) | 232 (43.9) |
| All other AA combinations | 24 (9.5) | 21 (7.6) | 45 (8.5) |
| | | | |
| **Landmark 2, progression-free survival** | **TMZ+RT**<br>177 (100) | **TMZ+RT+ marizomib**<br>196 (100) | **Total**<br>373 (100) |
| …..None | 92 (52.0) | 101 (51.5) | 193 (51.7) |
| All PA-PPI combinations | 74 (41.8) | 79 (40.3) | 153 (41.0) |
| All other AA combinations | 8 (4.5) | 12 (6.1) | 20 (5.4) |
| Not collected | 3 (1.7) | 4 (2.0) | 7 (1.9) |
| | | | |
| **Landmark 3, progression-free survival** | **TMZ+RT**<br>119 (100) | **TMZ+RT+ marizomib**<br>123 (100) | **Total**<br>242 (100) |
| …..None | 67 (56.3) | 60 (48.8) | 127 (52.5) |
| All PA-PPI combinations | 42 (35.3) | 47 (38.2) | 89 (36.8) |
| All other AA combinations | 6 (5.0) | 10 (8.1) | 16 (6.6) |
| Not collected | 4 (3.4) | 6 (4.9) | 10 (4.1) |
| | | | |
| **Landmark 1, overall survival** | **TMZ+RT**<br>342 (100) | **TMZ+RT+ marizomib**<br>353 (100) | **Total**<br>695 (100) |
| …..None | 149 (43.6) | 169 (47.9) | 318 (45.8) |
| All PA-PPI combinations | 163 (47.7) | 154 (43.6) | 317 (45.6) |
| All other AA combinations | 30 (8.8) | 30 (8.5) | 60 (8.6) |
| | | | |
| **Landmark 2, overall survival** | **TMZ+RT**<br>312 (100) | **TMZ+RT+ marizomib**<br>326 (100) | **Total**<br>638 (100) |
| …..None | 143 (45.8) | 167 (51.2) | 310 (48.6) |
| All PA-PPI combinations | 137 (43.9) | 125 (38.3) | 262 (41.1) |
| All other AA combinations | 24 (7.7) | 23 (7.1) | 47 (7.4) |
| Not collected | 8 (2.6) | 11 (3.4) | 19 (3.0) |
| | | | |
| **Landmark 3, overall survival** | **TMZ+RT**<br>290 (100) | **TMZ+RT+ marizomib**<br>307 (100) | **Total**<br>597 (100) |
| …..None | 141 (48.6) | 157 (51.1) | 298 (49.9) |
| All PA-PPI combinations | 91 (31.4) | 87 (28.3) | 178 (29.8) |
| All other AA combinations | 9 (3.1) | 18 (5.9) | 27 (4.5) |
| Not collected | 49 (16.9) | 45 (14.7) | 94 (15.7) |

Abbreviations: AA, anti-acids; ALDH1A1, aldehyde dehydrogenase 1 A1 (ALDH1A1); PA-PPI, potent ALDH1A1 activating PPI; PFS, progression-free survival; OS, overall survival; PPI proton pump inhibitor; RT, radiotherapy, TMZ, temozolomide.

© 2025 Le Rhun E et al. *JAMA Network Open*.

**eTable 11. Correlation between use of steroids and use of drugs of interest.**

| | None | | All PA-PPI | | AA without PA-PPI | |
|---|---|---|---|---|---|---|
| Steroid use, n (%) | no | yes | no | yes | no | Yes |
| | | | | | | |
| Baseline | 912 (79.4) | 236 (20.6) | 260 (36.5) | 453 (63.5) | 91 (32.3) | 191 (67.7) |
| | | | | | | |
| Progression-free survival | | | | | | |
| | | | | | | |
| Landmark 1 | 626 (56.2) | 488 (43.8) | 237 (22.9) | 798 (77.1) | 53 (15.9) | 281 (84.1) |
| | | | | | | |
| Landmark 2 | 431 (46.2) | 502 (53.8) | 150 (20.8) | 571 (79.2) | 29 (14.0) | 178 (86.0) |
| | | | | | | |
| Landmark 3 | 314 (43.5) | 408 (56.5) | 92 (20.9) | 348 (79.1) | 26 (17.7) | 121 (82.3) |
| | | | | | | |
| Overall survival | | | | | | |
| | | | | | | |
| Landmark 1 | 684 (55.4) | 551 (44.6) | 271 (22.3) | 944 (77.7) | 54 (14.2) | 326 (85.8) |
| | | | | | | |
| Landmark 2 | 565 (45.0) | 691 (55.0) | 194 (18.6) | 850 (81.4) | 39 (12.8) | 265 (87.2) |
| | | | | | | |
| Landmark 3 | 534 (42.1) | 733 (57.9) | 148 (18.5) | 653 (81.5) | 32 (13.7) | 202 (86.3) |

Abbreviations: AA, anti-acids; ALDH1A1, aldehyde dehydrogenase 1 A1 (ALDH1A1); PA-PPI, potent ALDH1A1 activating PPI; PPI proton pump inhibitor.

© 2025 Le Rhun E et al. *JAMA Network Open*.

## eFigure 1. CONSORT diagram

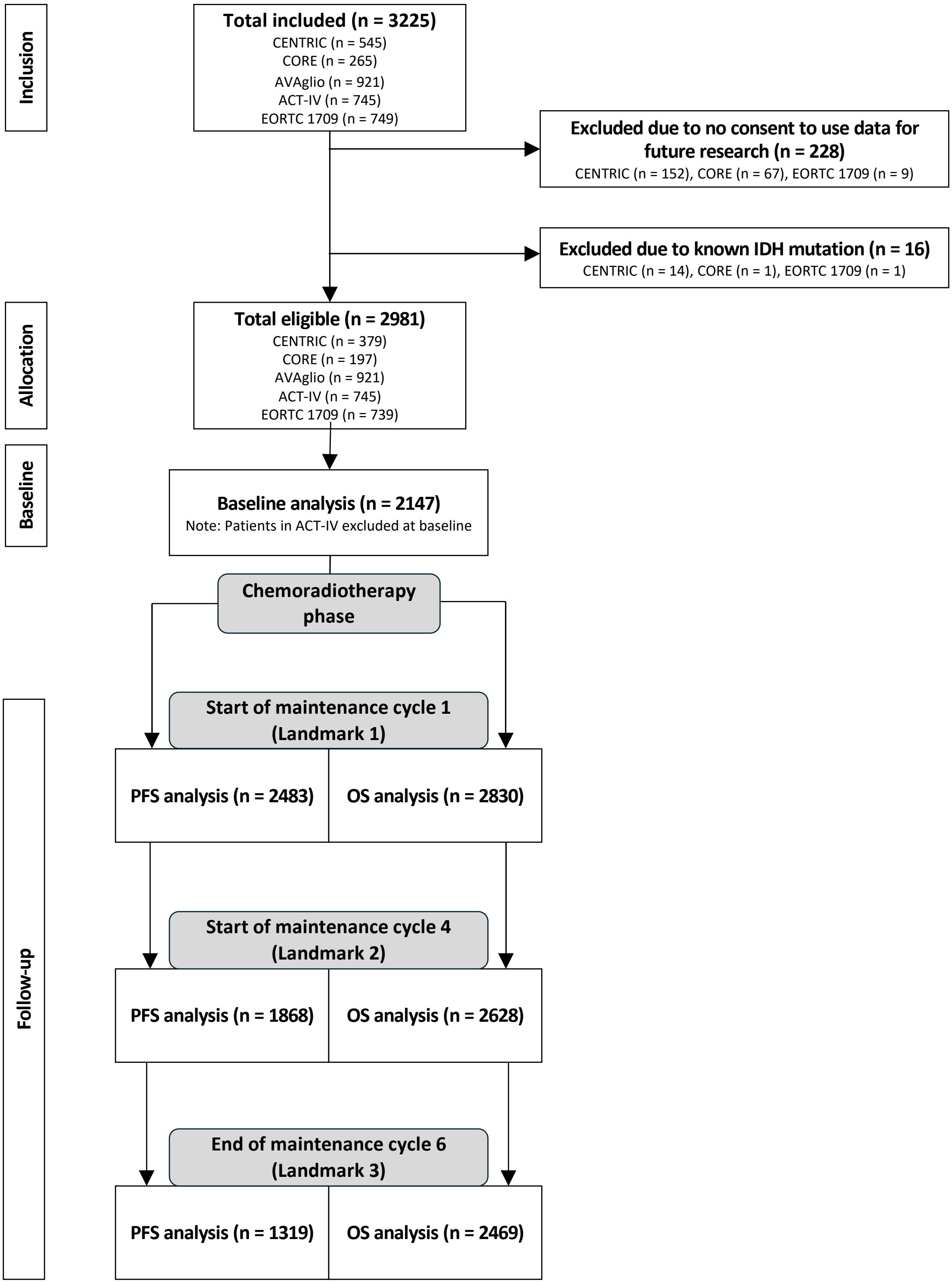


Abbreviations: IDH, isocitrate dehydrogenase; PFS, progression-free survival; OS, overall survival.

© 2025 Le Rhun E et al. *JAMA Network Open*.

**eFigure 2. Associations of the use of PA-PPI with progression-freel survival outcome in patients with newly diagnosed glioblastoma.** Progression-free survival (PFS) (A,B,C,D) by use of PPI with potent activation (PA) of ALDH1A1 (PA-PPI) at study entry (baseline) (A) and at defined landmarks: start of maintenance cycles 1 (landmark 1) (B) and 4 (landmark 2) (C), and end of maintenance cycle 6 (landmark 3) (D).

**A**

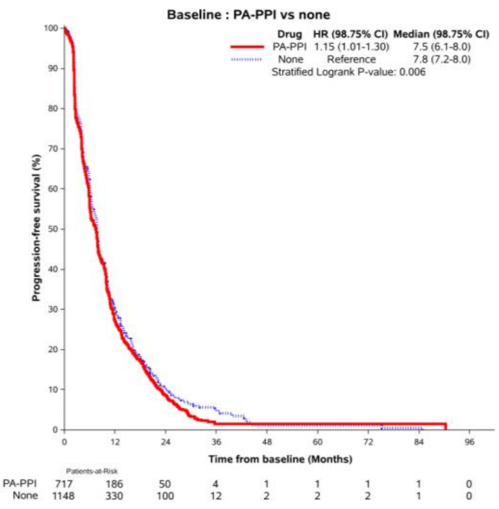


**B**

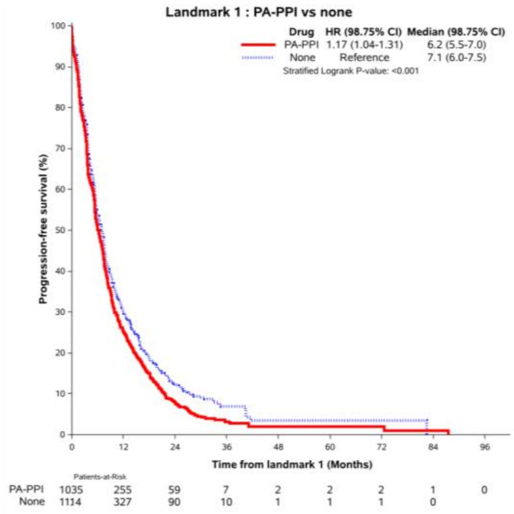


**C**

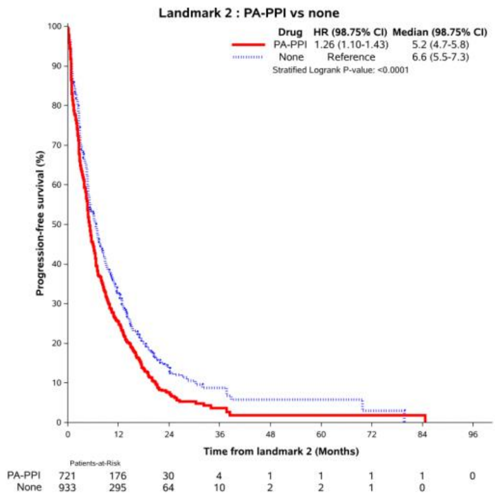


**D**

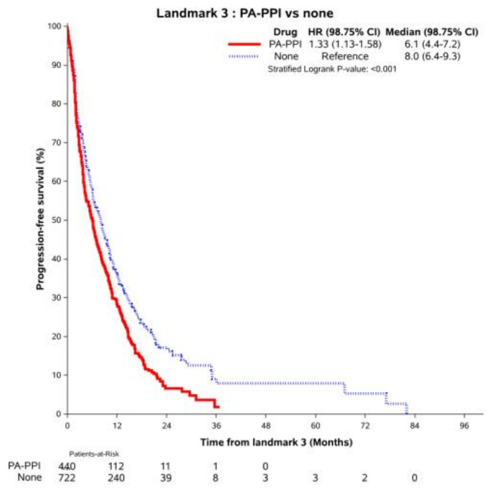


© 2025 Le Rhun E et al. *JAMA Network Open*.

**eFigure 3. Associations of anti-acids (AA) with or without weak ALDH1A1-activating PPI use with outcome in patients with newly diagnosed glioblastoma.** Progression-free survival (A,C,E,G) and overall survival (OS) (B,D,F,H) by use of AA with or without weak ALDH1A1-activating PPI use at study entry (baseline) (A,B) and at defined landmarks: start of maintenance cycles 1 (landmark 1) (C,D) and 4 (landmark 2) (E,F), and end of maintenance cycle 6 (landmark 3) (G,H).

A

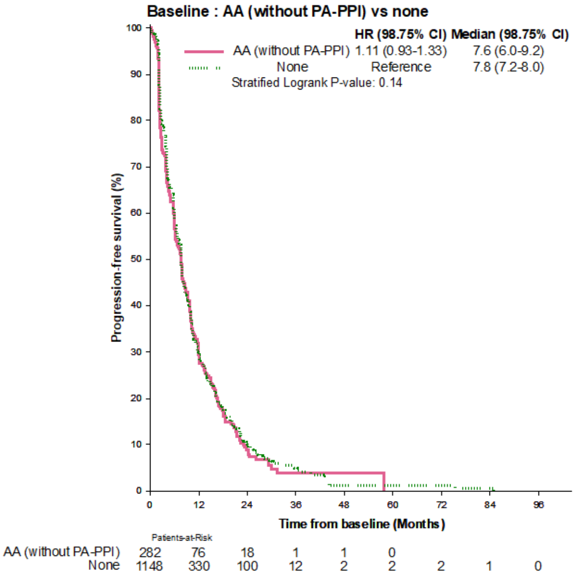


B

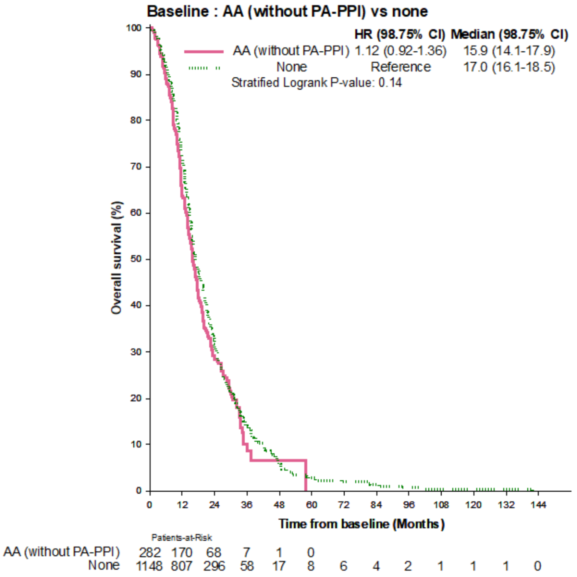


C

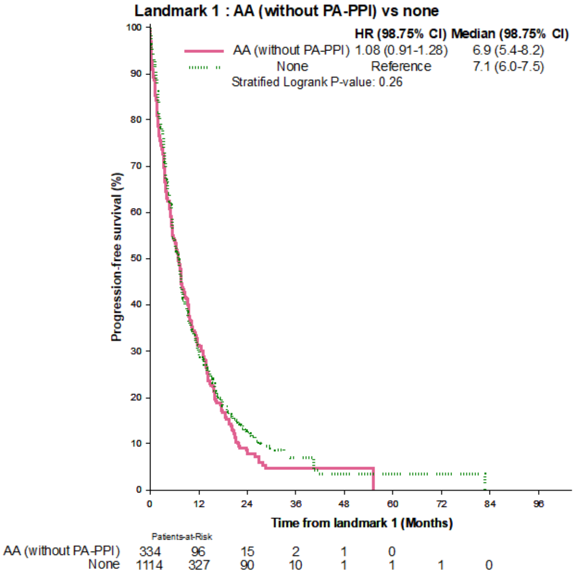


D

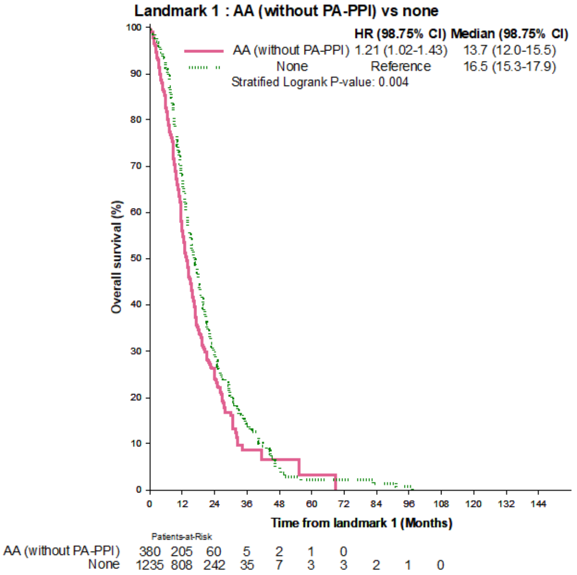


© 2025 Le Rhun E et al. *JAMA Network Open*.

E

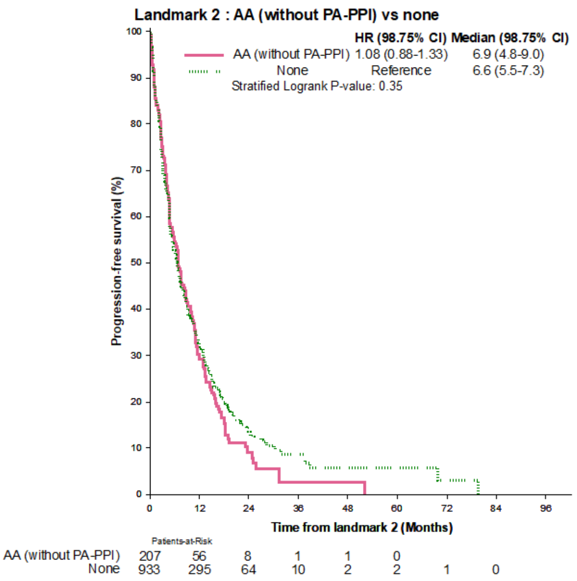


F

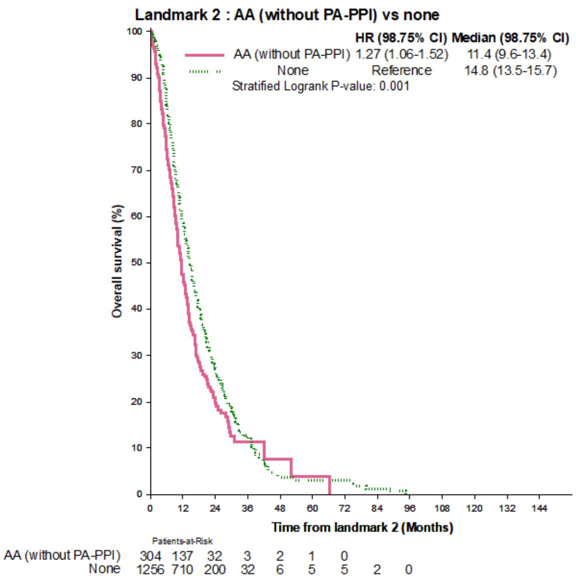


G

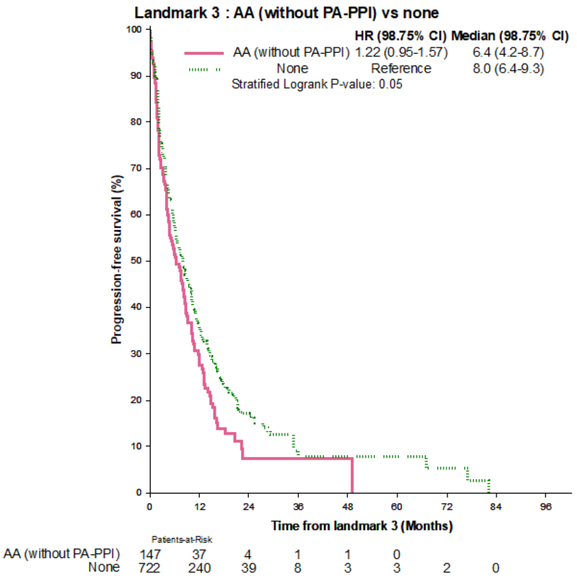


H

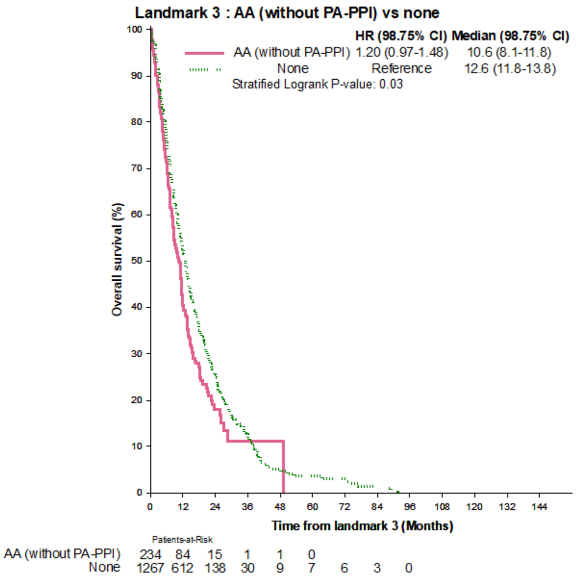


© 2025 Le Rhun E et al. *JAMA Network Open*.

**eFigure 4. Associations of PA-PPI use with outcome in patients with newly diagnosed glioblastoma stratified by O$^6$-methylguanine DNA methyltransferase *(MGMT)* promoter methylation status at landmark 1.** Treatment effect refers to outcome associations of PPI intake. PFS, progression-free survival; OS, overall survival.

**A**

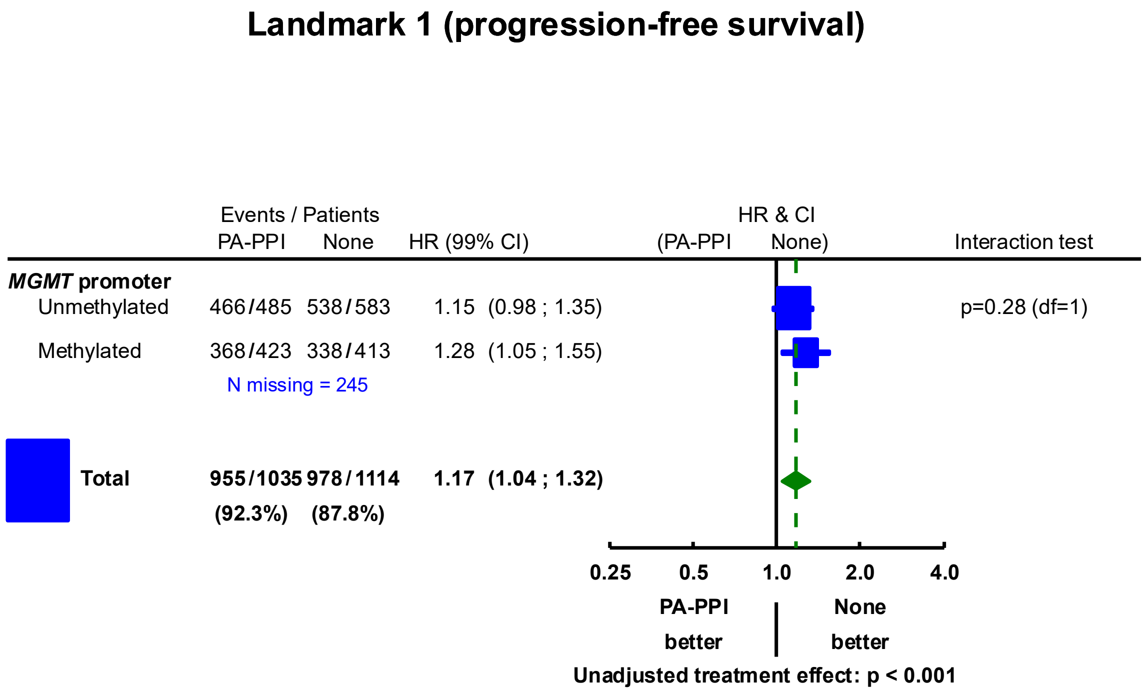


**B**

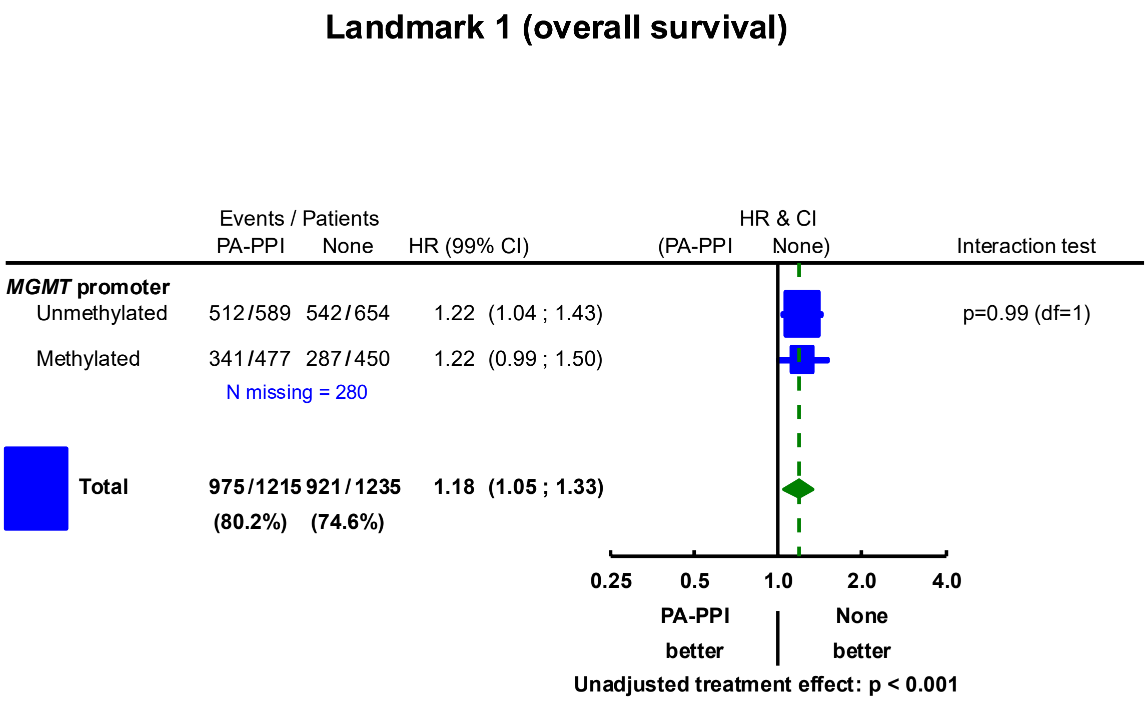


© 2025 Le Rhun E et al. *JAMA Network Open*.

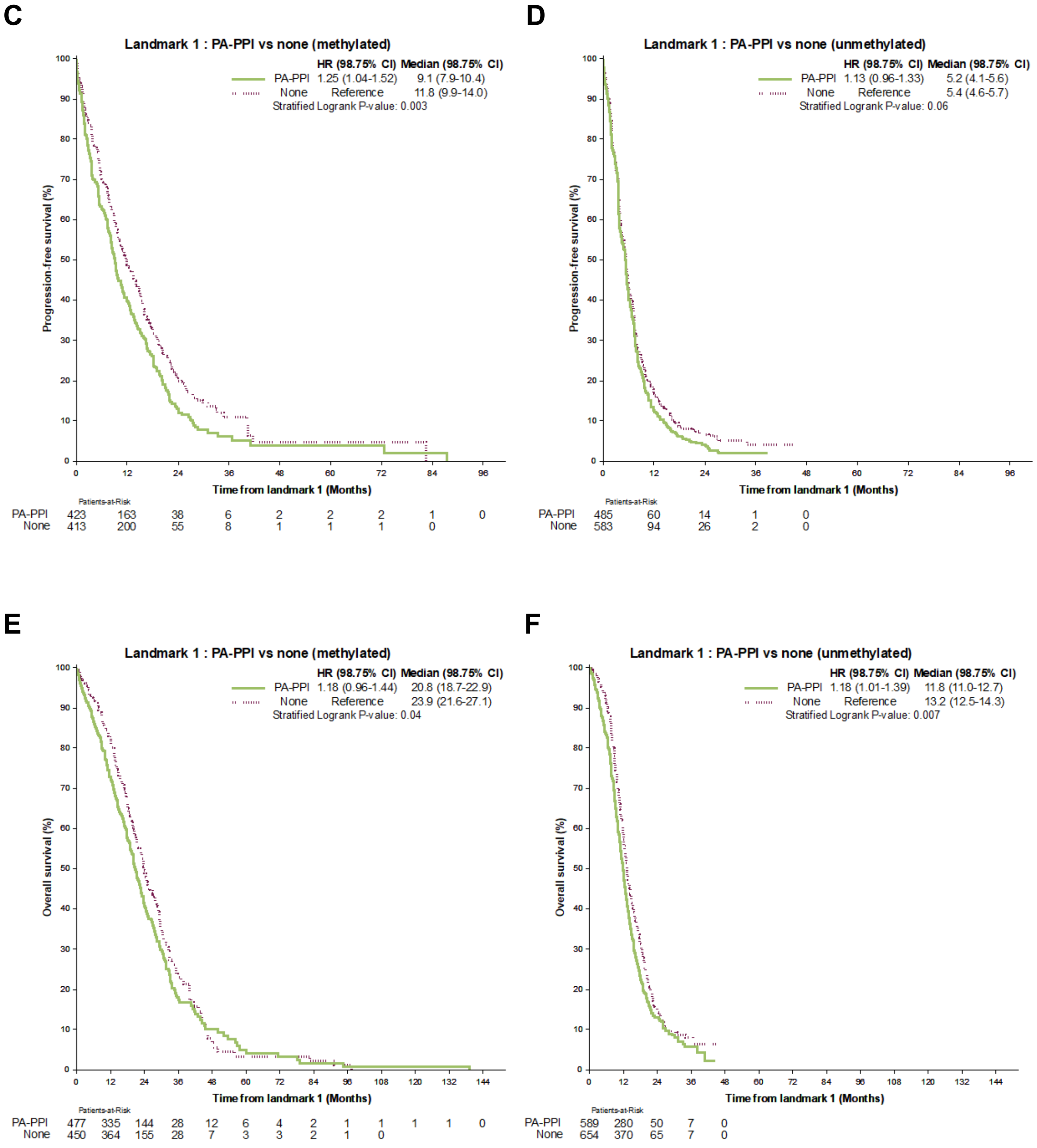


© 2025 Le Rhun E et al. *JAMA Network Open*.

**eFigure 5. Associations of PA-PPI use with outcome in patients with newly diagnosed glioblastoma assigned to the control groups of the five trials, stratified by O$^{6}$-methylguanine DNA methyltransferase *(MGMT)* promoter methylation status at landmark 2.** A, B, Forest plots for progression-free and overall survival. C-F, survival curves for progression-free and overall survival.

**A**

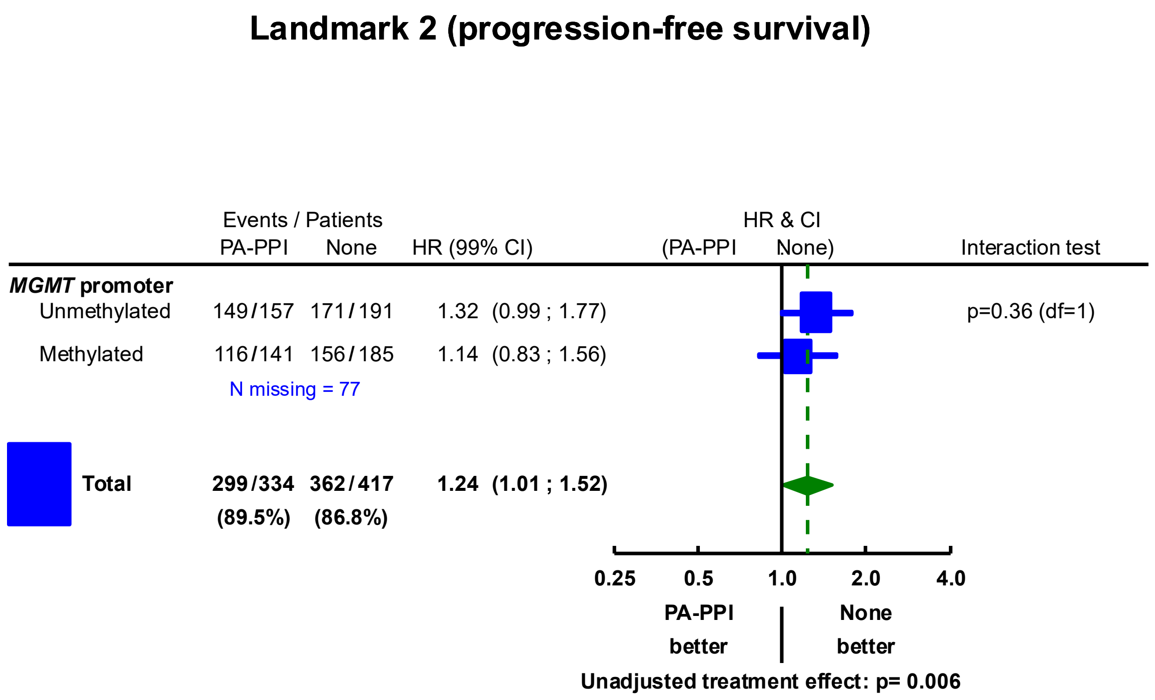


**B**

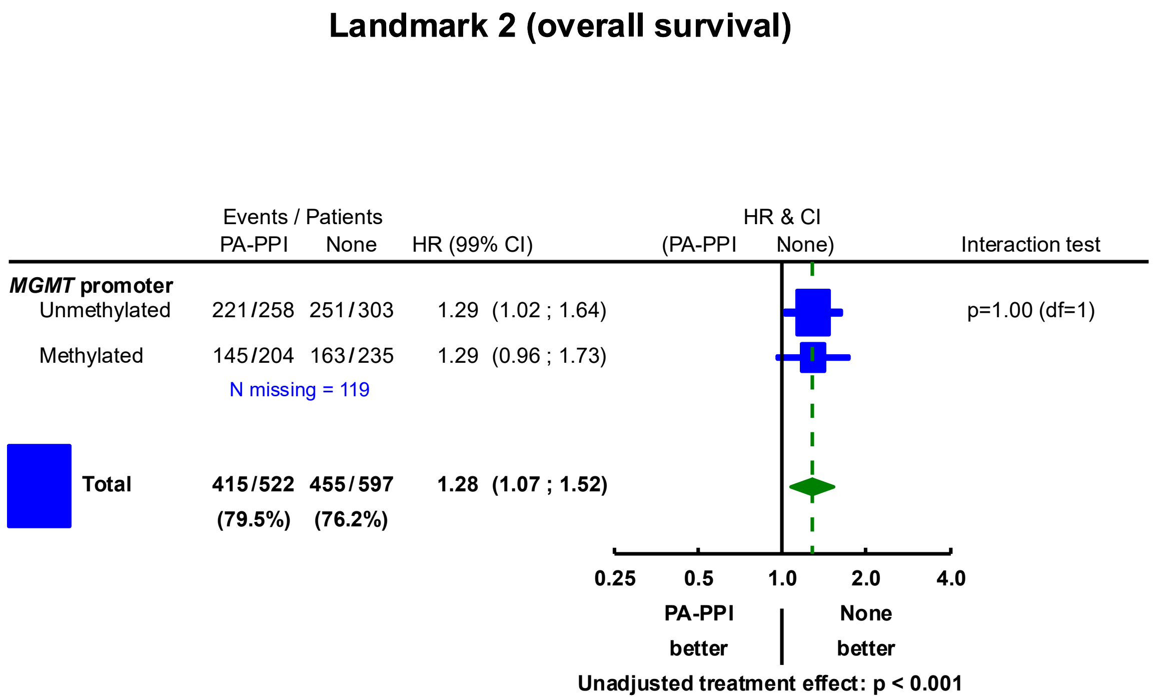


© 2025 Le Rhun E et al. *JAMA Network Open*.

**C**

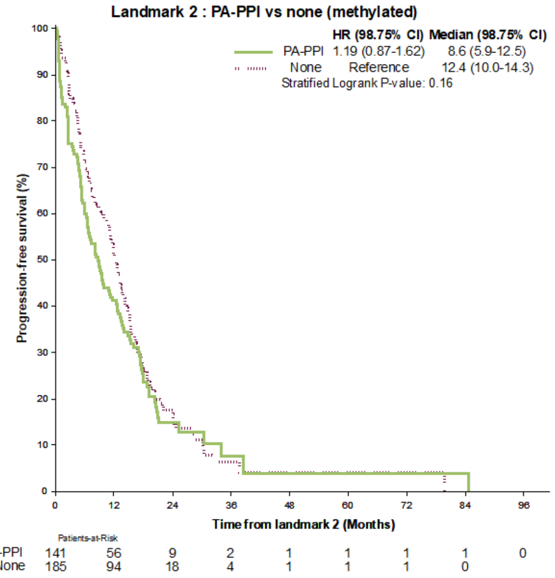


**D**

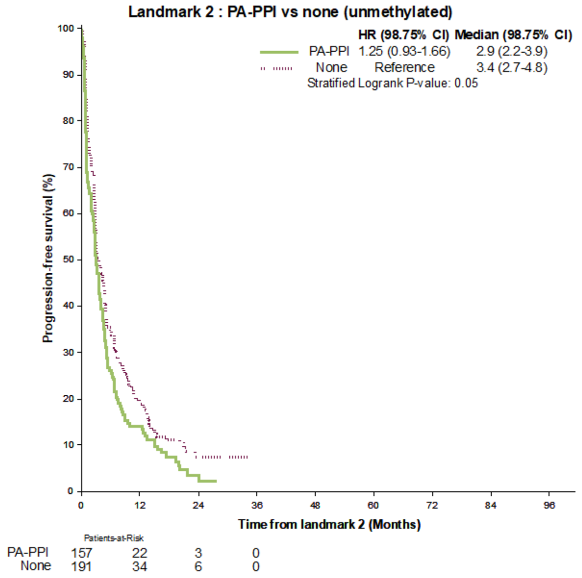


**E**

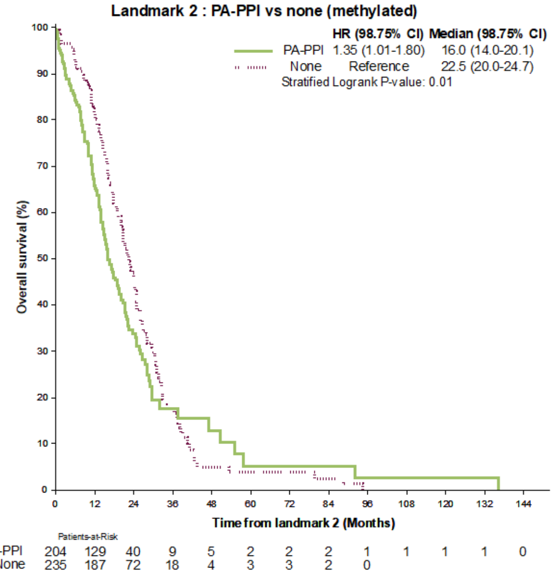


**F**

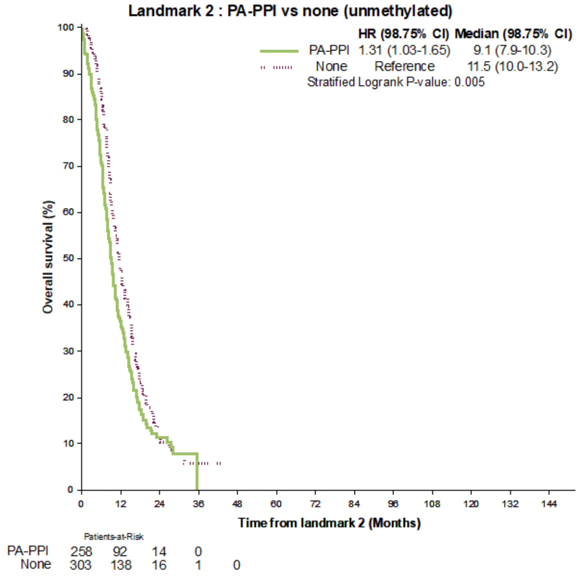


© 2025 Le Rhun E et al. *JAMA Network Open*.

**eFigure 6. Interaction of steroid and PA-PPI use for progression-free survival associations (Forest plots).** Treatment effect refers to outcome associations of PPI intake at study entry (baseline) (A) and at defined landmarks: start of maintenance cycles 1 (landmark 1) (B) and 4 (landmark 2) (C), and end of maintenance cycle 6 (landmark 3) (D).

**A**

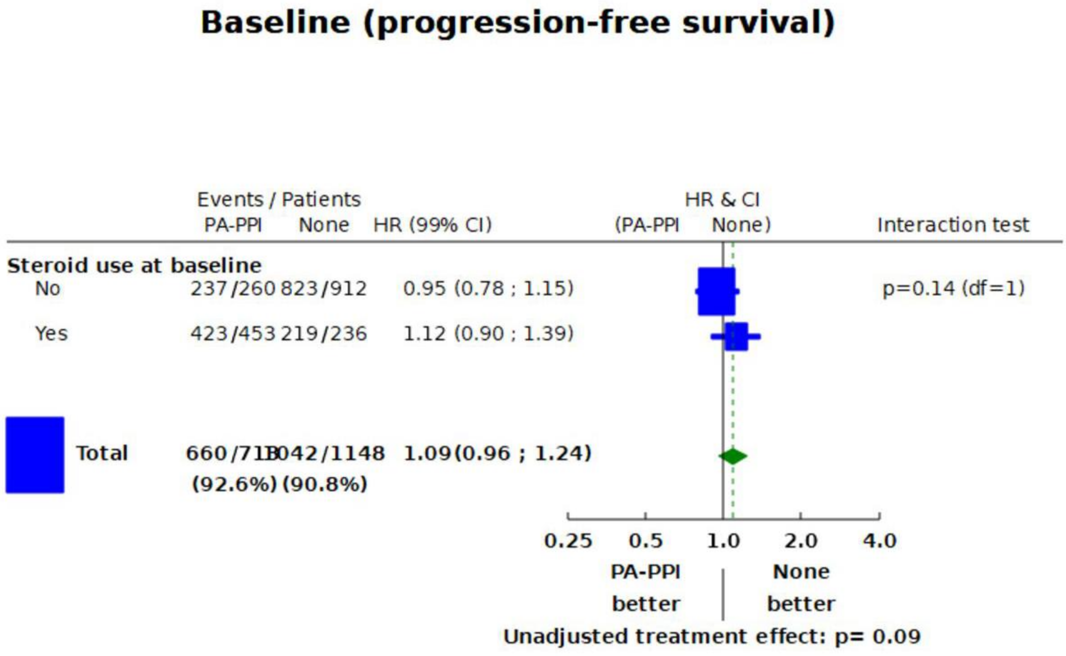


**B**

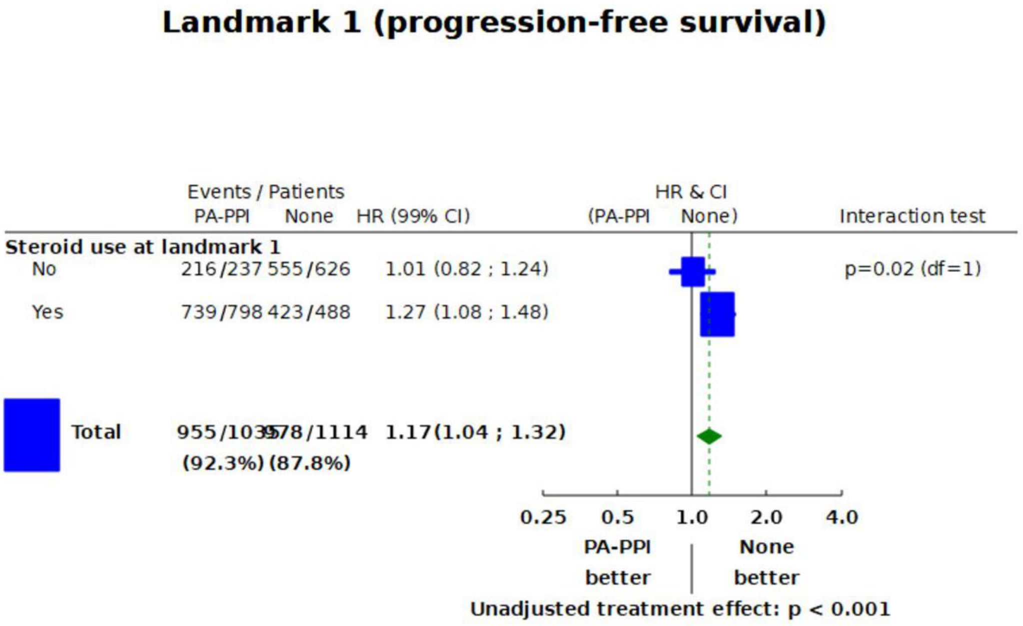


© 2025 Le Rhun E et al. *JAMA Network Open*.

C

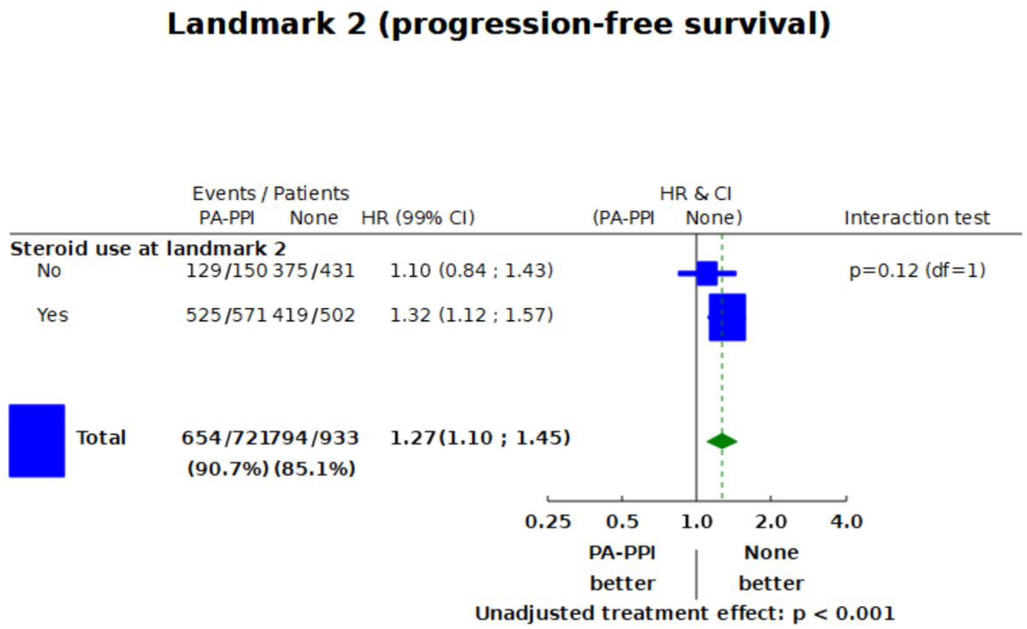


D

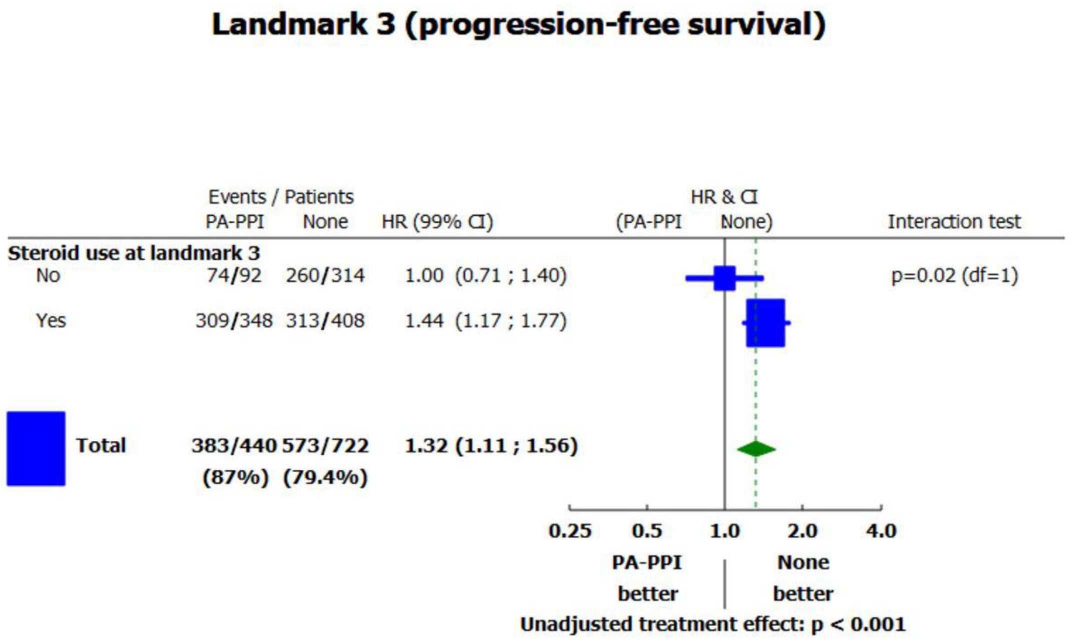


© 2025 Le Rhun E et al. *JAMA Network Open*.

**eFigure 7. Interaction of steroid and AA (without PA-PPI) use for outcome associations (Forest plots).** A, C, E, G, progression-free survival, B, D, F, H, overall survival. Treatment effect refers to outcome associations of non-PPI AA intake.

**A**

## Baseline (progression-free survival)

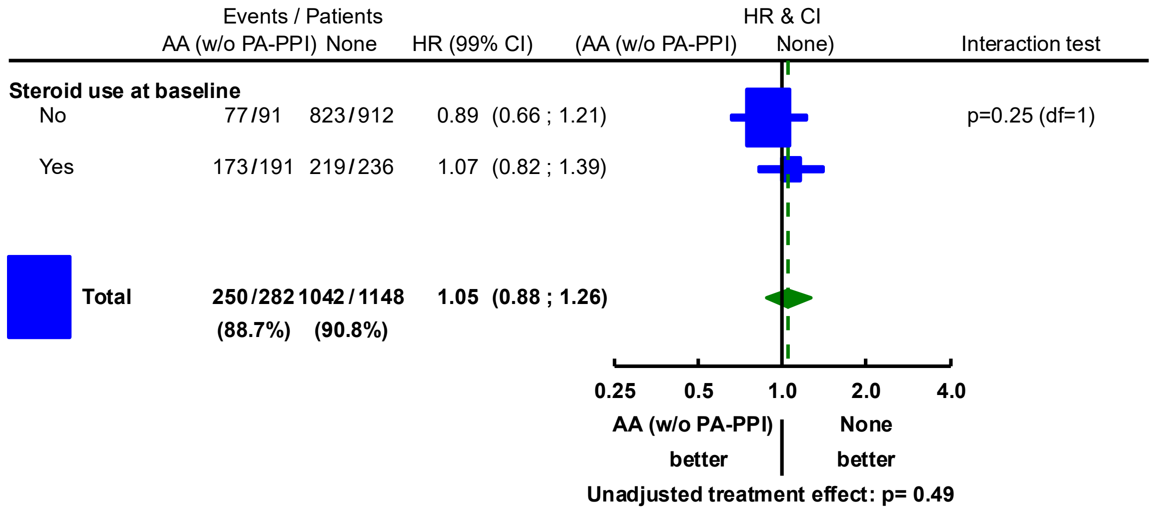


**B**

## Baseline (overall survival)

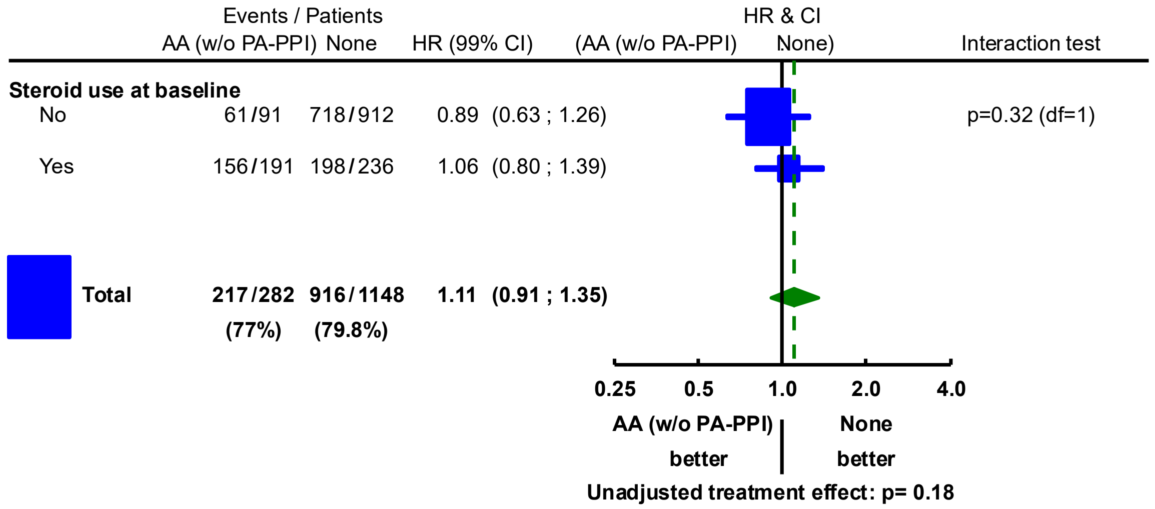


© 2025 Le Rhun E et al. *JAMA Network Open*.

C

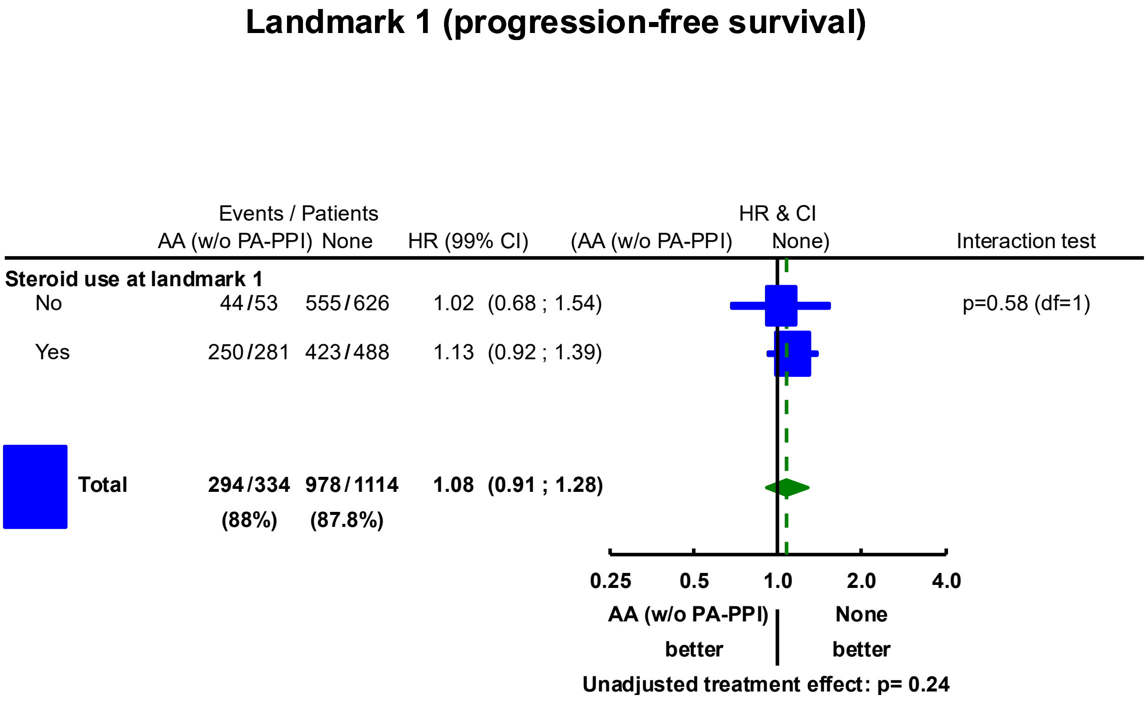


D

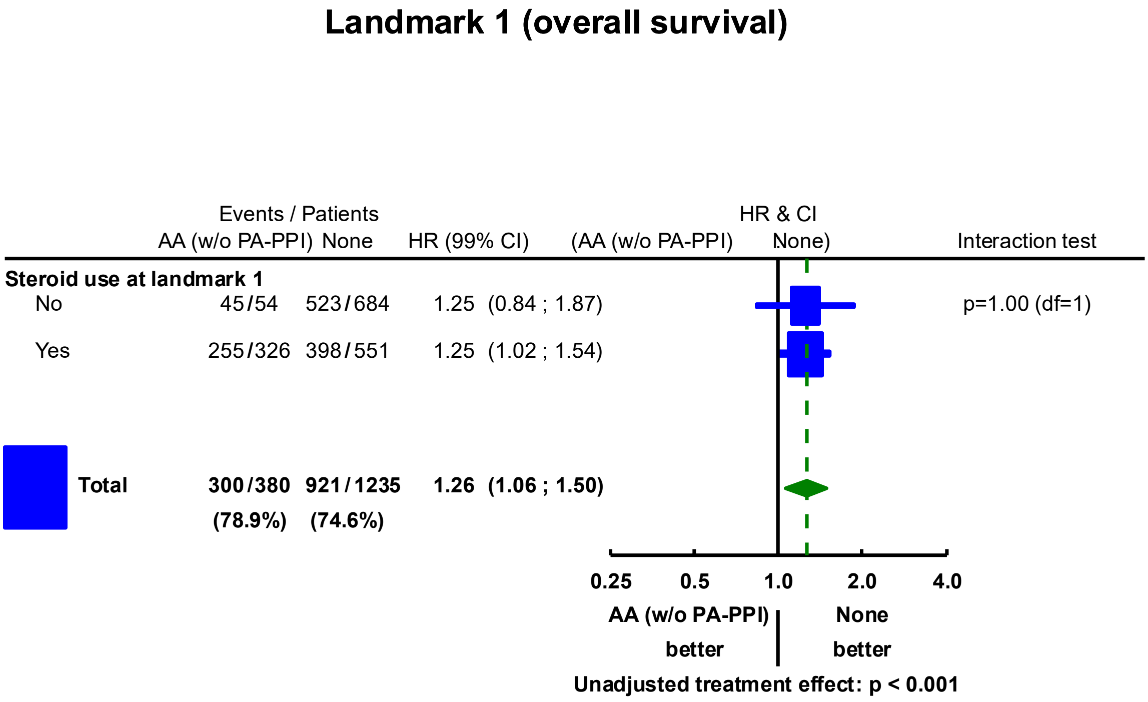


© 2025 Le Rhun E et al. *JAMA Network Open*.

**E**

## Landmark 2 (progression-free survival)

| | Events / Patients AA (w/o PA-PPI) | Events / Patients None | HR (99% CI) | Interaction test |
|---|---|---|---|---|
| **Steroid use at landmark 2** | | | | |
| No | 25/29 | 375/431 | 0.93 (0.54 ; 1.58) | p=0.39 (df=1) |
| Yes | 153/178 | 419/502 | 1.13 (0.88 ; 1.44) | |
| **Total** | **178/207 (86%)** | **794/933 (85.1%)** | **1.09 (0.88 ; 1.35)** | |

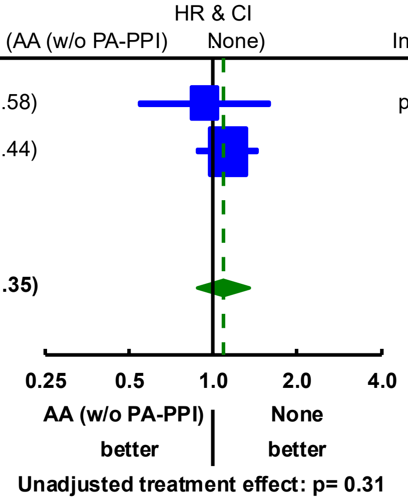


**F**

## Landmark 2 (overall survival)

| | Events / Patients AA (w/o PA-PPI) | Events / Patients None | HR (99% CI) | Interaction test |
|---|---|---|---|---|
| **Steroid use at landmark 2** | | | | |
| No | 33/39 | 435/565 | 1.33 (0.83 ; 2.11) | p=0.76 (df=1) |
| Yes | 204/265 | 499/691 | 1.25 (1.01 ; 1.55) | |
| **Total** | **237/304 (78%)** | **934/1256 (74.4%)** | **1.32 (1.09 ; 1.59)** | |

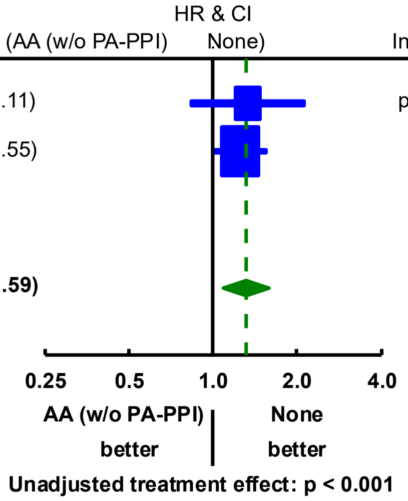


© 2025 Le Rhun E et al. *JAMA Network Open*.

G

## Landmark 3 (progression-free survival)

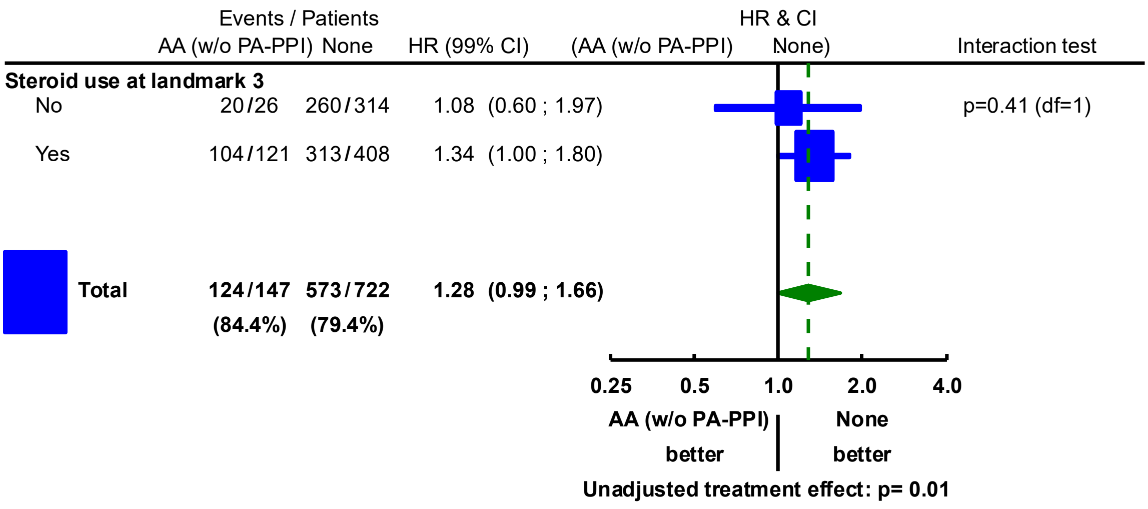


H

## Landmark 3 (overall survival)

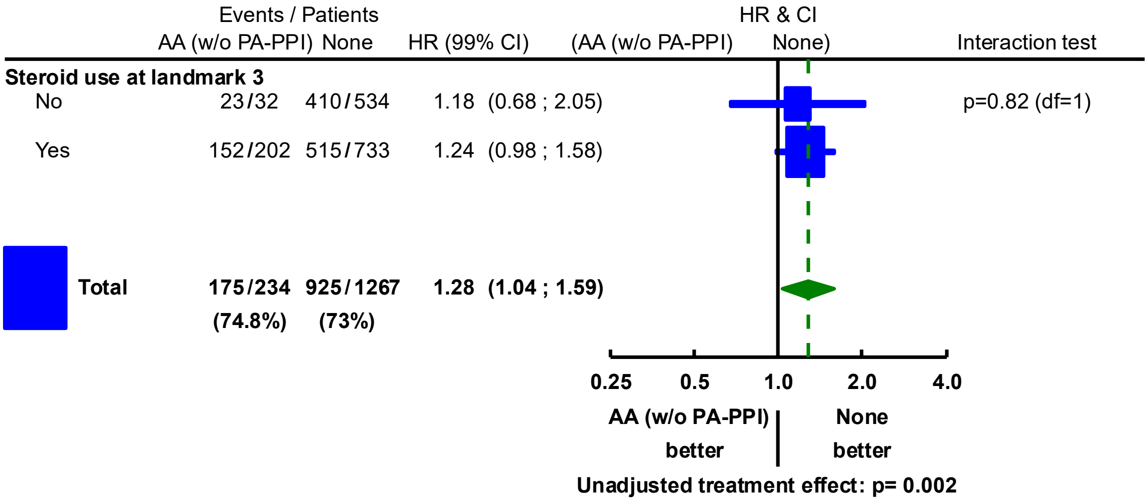


© 2025 Le Rhun E et al. *JAMA Network Open*.

**eAppendix 1: Details of multiple imputation.**

In some trials, the collection of concomitant medication data was stopped after the end of treatment or disease progression. Since the missing data is directly related to the patient's treatment or progression status, the mechanism of missingness may be informative. In such cases, a complete case (CC) analysis, where only patients with complete data are included, excluding those with missing drug information, may lead to potentially biased estimates.
To investigate the impact of the missing data mechanism on the analysis, we compared the results from the CC analysis with multiple imputation (MI) techniques. Imputation is a statistical method that replaces missing values with estimated values based on the observed data. The steps of multiple imputation used in our analysis were as follows:

1. Logistic regression models with Fully Conditional Specification (FCS) were implemented to generate multiple complete data sets based on the available information (drug categories, age, sex, steroid, MGMT, WHO-PS, and extent of surgery).
2. Multivariate Cox models were fitted for each complete data set to estimate the model parameters.
3. Rubin's rule was applied to combine results from all Cox analyses, producing a single set of parameter estimates. This method accounts for both variability in the Cox models and the uncertainty due to the missing data.

Consistency in results and conclusions between the CC and MI analyses suggest that the missingness mechanism may not introduce significant bias into the results.

**eAppendix 2: PPI and ALDH1**

High levels of ALDH1 kept glioma cells in an undifferentiated, stem cell-like state indicated by the low expression of beta-III-tubulin. Conversely, inhibition of ALDH1 decreased number and size of glioblastoma neurospheres, and cell lines lacking ALDH1 did not form tumor spheroids (7). Similarly, ALDH1A1 expression predicted temozolomide resistance of glioma cell lines *in vitro*, and sensitivity of ALDH1A1 positive and *MGMT*-expressing cells to temozolomide was restored by inhibition of ALDH1A1 by 4-diethylaminobenzaldehyde or by sh-RNA-mediated knockdown of ALDH1A1 (8). Only glioblastoma cells with high ALDH1 expression were able to restore heterogeneous populations whereas cells with low ALDH1 levels could not when tested for asymmetric division. The capacity of cells with low ALDH1 levels to divide asymmetrically into cells with either high or low ALDH1 expression was restored after exposure to hypoxic culture conditions (9). Rare ALDH1A1-expressing glioblastoma cells have been proposed to enrich and acquire AKT-mediated drug resistance in response to temozolomide exposure (10). ALDH1A1 levels were also proposed as a biomarker of glioblastoma cells with high invasive potential, altered oxidative stress and resistance to inhibitor of epidermal growth factor receptor (11).
ADLH1A1 expression has been linked to higher WHO grade of gliomas (12) and associated with inferior outcome among patients with glioblastoma (8). Yet, both the link of ALDH1A1 to stemness and its negative prognostic value have not been consistently reproduced (13).

© 2025 Le Rhun E et al. *JAMA Network Open*.

**eReferences**


1. Weller M, Butowski N, Tran DD, et al. Rindopepimut with temozolomide for patients with newly diagnosed, EGFRvIII-expressing glioblastoma (ACT IV): a randomised, double-blind, international phase 3 trial. *Lancet Oncol*. 2017;18(10):1373-1385. doi:10.1016/S1470-2045(17)30517-X

2. Chinot OL, Wick W, Mason W, et al. Bevacizumab plus radiotherapy-temozolomide for newly diagnosed glioblastoma. *N Engl J Med*. 2014;370(8):709-722. doi:10.1056/NEJMoa1308345

3. Stupp R, Hegi ME, Gorlia T, et al. Cilengitide combined with standard treatment for patients with newly diagnosed glioblastoma with methylated MGMT promoter (CENTRIC EORTC 26071-22072 study): a multicentre, randomised, open-label, phase 3 trial. *Lancet Oncol*. 2014;15(10):1100-1108. doi:10.1016/S1470-2045(14)70379-1

4. Nabors LB, Fink KL, Mikkelsen T, et al. Two cilengitide regimens in combination with standard treatment for patients with newly diagnosed glioblastoma and unmethylated MGMT gene promoter: results of the open-label, controlled, randomized phase II CORE study. *Neuro-oncology*. 2015;17(5):708-717. doi:10.1093/neuonc/nou356

5. Roth P, Gorlia T, Reijneveld JC, et al. Marizomib for patients with newly diagnosed glioblastoma: A randomized phase 3 trial. *Neuro Oncol*. 2024;26(9):1670-1682. doi:10.1093/neuonc/noae053

6. Calleja LF, Belmont-Díaz JA, Medina-Contreras O, et al. Omeprazole as a potent activator of human cytosolic aldehyde dehydrogenase ALDH1A1. *Biochim Biophys Acta Gen Subj*. 2020;1864(1):129451. doi:10.1016/j.bbagen.2019.129451

7. Rasper M, Schäfer A, Piontek G, et al. Aldehyde dehydrogenase 1 positive glioblastoma cells show brain tumor stem cell capacity. *Neuro Oncol*. 2010;12(10):1024-1033. doi:10.1093/neuonc/noq070

8. Schäfer A, Teufel J, Ringel F, et al. Aldehyde dehydrogenase 1A1--a new mediator of resistance to temozolomide in glioblastoma. *Neuro Oncol*. 2012;14(12):1452-1464. doi:10.1093/neuonc/nos270

9. Soehngen E, Schaefer A, Koeritzer J, et al. Hypoxia upregulates aldehyde dehydrogenase isoform 1 (ALDH1) expression and induces functional stem cell characteristics in human glioblastoma cells. *Brain Tumor Pathol*. 2014;31(4):247-256. doi:10.1007/s10014-013-0170-0

10. Kebir S, Ullrich V, Berger P, et al. A Sequential Targeting Strategy Interrupts AKT-Driven Subclone-Mediated Progression in Glioblastoma. *Clin Cancer Res*. 2023;29(2):488-500. doi:10.1158/1078-0432.CCR-22-0611

11. McKinney A, Lindberg OR, Engler JR, et al. Mechanisms of Resistance to EGFR Inhibition Reveal Metabolic Vulnerabilities in Human GBM. *Mol Cancer Ther*. 2019;18(9):1565-1576. doi:10.1158/1535-7163.MCT-18-1330

12. Xu SL, Liu S, Cui W, et al. Aldehyde dehydrogenase 1A1 circumscribes high invasive glioma cells and predicts poor prognosis. *Am J Cancer Res*. 2015;5(4):1471-1483.

13. Adam SA, Schnell O, Pöschl J, et al. ALDH1A1 is a marker of astrocytic differentiation during brain development and correlates with better survival in glioblastoma patients. *Brain Pathol*. 2012;22(6):788-797. doi:10.1111/j.1750-3639.2012.00592.x


© 2025 Le Rhun E et al. *JAMA Network Open*.
