# Supplementary material for: Proton Pump Inhibitor Use and Survival in Patients With Newly Diagnosed Glioblastoma
Source: JAMA Netw Open. 2025 Nov 25;8(11):e2545578. doi: 10.1001/jamanetworkopen.2025.45578 (PMC12648345; doi:10.1001/jamanetworkopen.2025.45578)
Supplement: Supplement 2. — Data Sharing Statement [file jamanetwopen-e2545578-s002.pdf]

Data Sharing Statement

Le Rhun. Proton Pump Inhibitor Use and Survival in Patients With Newly Diagnosed Glioblastoma. *JAMA Netw Open*. Published November 25, 2025.
doi:10.1001/jamanetworkopen.2025.45578

Data
Data available: Yes
Data types: Deidentified participant data
How to access data: request has to be sent to EORTC
When available: With publication

Supporting Documents
Document types: None

Additional Information
Who can access the data: pending EORTC decision
Types of analyses: pending EORTC decision
Mechanisms of data availability: pending EORTC decision
